# Naltrexone blocks alcohol-induced effects on kappa-opioid receptors in the plasma membrane

**DOI:** 10.1038/s41398-024-03172-8

**Published:** 2024-11-24

**Authors:** Sho Oasa, Erdinc Sezgin, Yuelong Ma, David A. Horne, Mihajlo D. Radmilović, Tijana Jovanović-Talisman, Rémi Martin-Fardon, Vladana Vukojević, Lars Terenius

**Affiliations:** 1 Department of Clinical Neuroscience, Center for Molecular Medicine, Karolinska Institutet, Stockholm, SE-17176 Sweden; 2 Science for Life Laboratory, Department of Women’s and Children’s Health, Karolinska Institutet, Stockholm, SE-17165 Sweden; 3 Synthetic Biopolymer Chemistry Core, Beckman Research Institute; City of Hope, 1500 East Duarte Road, Duarte, CA 91010 USA; 4 Department of Cancer Biology and Molecular Medicine, Beckman Research Institute, City of Hope, 1500 East Duarte Road, Duarte, CA 91010 USA; 5 Institute of Physics Belgrade, University of Belgrade, Pregrevica 118, Belgrade, 11080 Serbia; 6 Department of Molecular Medicine, The Scripps Research Institute, La Jolla, CA 92037 USA

**Keywords:** Pharmacology, Molecular neuroscience, Addiction

## Abstract

Naltrexone (NTX), a homolog of the opiate antidote naloxone, is an orally active long-acting general opioid receptor antagonist used in the treatment of opiate dependence. NTX is also found to relieve craving for alcohol and is one of few FDA-approved medications for treatment of alcohol use disorder (AUD). While it was early on established that NTX acts by blocking the binding of endogenous opioid peptide ligands released by alcohol, experimental evidence emerged that could not be fully accounted for by this explanation alone, suggesting that NTX may have additional modes of action. Mu- and kappa-opioid receptors (MOP and KOP, respectively) are structurally related G-protein-coupled receptors (GPCRs), but they are anatomically differently distributed and functionally distinct, often mediating opposite responses, with MOP typically promoting euphoria and reward, while KOP is associated with dysphoria and aversive states. While the actions of NTX on MOP are extensively characterized, the interactions with KOP are not. Here, we used sensitive fluorescence-based methods with single-molecule sensitivity to study in live cells the influence of alcohol (ethanol, EtOH) on KOP and the interaction between KOP and NTX. Our data show that alcohol, at relevant concentrations (10–40 mM), alters KOP interactions with the lipid environment in the plasma membrane. The counteracting effects of NTX are exerted by both its canonical action on KOP and its hitherto unrevealed effects on the lateral dynamics and organization of lipids in the plasma membrane. The KOP-specific antagonist LY2444296, in clinical trial for major depressive disorder (MDD), blocks KOP but does not show the full action profile of NTX. The therapeutic effect of NTX treatment in AUD may in part be due to direct actions on KOP and in part due to its effect on the surrounding lipid environment.

## Introduction

Social drinking, i.e. moderate consumption of alcohol (ethanol (EtOH)) gives a sensation of elatedness and relaxation. This is very different from the drive to binge drinking, to get intoxicated. While the euphoric effects of alcohol, primarily mediated through the mu-opioid receptor (MOP), have been well studied, much less is known about the effects mediated by the kappa-opioid receptor (KOP) that contribute to alcohol abuse. We have decided to assess at the cellular and molecular level actions of alcohol on KOP and its interactions with naltrexone (NTX), which is approved by the Food and Drug Administration (FDA) for the treatment of alcohol use disorder (AUD). Recently, NTX has been advocated for the treatment of alcohol misuse as “one of the most underutilized interventions in medicine” [1].

Early experimental studies reporting the potential therapeutic effects of NTX in alcohol dependence [2, 3] were followed by clinical studies in human subjects showing that NTX reduces the feeling of “high” induced by alcohol in alcohol-dependent individuals [4]. NTX was early-on shown to counteract alcohol-induced release of endogenous opioids (enkephalins and β-endorphin) acting on MOP [5, 6]. However, the response was not universal and it has been suggested that differences in response may be genetically determined. The potential therapeutic effects of NTX for the treatment of AUD eventually led to an FDA approval for this indication, which is significant since very few medications for this condition are available.

It is commonly assumed that AUD is a consequence of the euphoriant activity of alcohol (positive reinforcement) and of craving, the urge to resume consumption in abstinence (negative reinforcement). Positive reinforcement is mainly exerted via MOP-mediated pathways, whereas negative reinforcement is mainly exerted via KOP-mediated pathways [7]. Classic binding analysis has shown that NTX primarily binds to MOP and has lower affinity for KOP. This has been an opening for the clinical use of compounds with overlapping affinities for MOP and KOP, such as pentazocine or buprenorphine. Our previous studies have shown that NTX has a significant influence on ethanol-induced effects on KOP lateral organization in the plasma membrane [8]. Using sensitive fluorescence microscopy imaging and correlation spectroscopy technologies, we have observed that ethanol in pharmacologically relevant concentrations, 10–40 mM, affects glycosylphosphatidylinositol-enriched membrane domains and MOP- and KOP-harboring nanoscale clusters; and that these effects are largely blocked by NTX [8]. Nanoscale cluster formation is a common feature of plasma membrane receptors that is necessary for their functions [9–11], in particular agonist-activated receptors are shown to be prone to form nanoscale clusters, probably also forming homodimers *via* the modulation of local receptor density [12]. Our previous studies suggest that ethanol modulates the functions of MOP and KOP *via* their cluster formation and dimerization capacity. This may also be an underlying molecular mechanism for the KOP supersensitivity observed in a behavioral/neurochemistry analysis in mice [13].

An asset for drug development is the reported structural characterization of the dynorphin/KOP system. The X-ray structure of KOP with the antagonist JDTic was one of the first in the opioid receptor family [14]. The dynamics of the interaction between dynorphin and KOP were followed using nuclear magnetic resonance (NMR) [15]. Membrane lipids have been seen as catalysts for dynorphin-KOP interactions: ligand accumulation at the plasma membrane by electrostatic attraction and direct ligand-plasma membrane lipid interaction result in lower energy needed for the ligand to bind to the receptor [16]. This is possibly also relevant for NTX-KOP interactions: under normal physiology (pH 7.4), NTX is neutral/protonated (literature findings for the acid dissociation constant of NTX vary, 7.5 ≤ pK_a,NTX_ ≤ 8.6), and moderately lipophilic (partition coefficient Log P = 1.76 for n-octanol/buffer pH 7.4 at 37 °C). Ethanol-induced reduction of the dielectric constant of the surrounding water medium can affect the interplay between electrostatic and lipophilic interactions, thus affecting NTX partitioning into the lipid bilayer and NTX-KOP interactions.

In this work, we used cells genetically modified to express KOP fused with the enhanced green fluorescent protein (KOP-eGFP) and employed fluorescence lifetime imaging microscopy (FLIM) to quantitatively characterize in live cells EtOH effects on KOP-eGFP in the plasma membrane by measuring eGFP fluorescence lifetime (FL). FL, i.e., the lifetime of a fluorescent molecule in the excited state, is an immanent property of the fluorescent molecule that neither depends on the concentration, nor on the laser intensity used for its excitation, nor on photobleaching of the fluorescent molecule. It is, however, sensitive to changes in the immediate environment, such as changes in temperature [17], pH [18], molecular crowding [19], protein oligomerization [20] /aggregation [21]. Although FLIM is most often integrated with Förster resonance energy transfer (FLIM-FRET) to assess molecular interactions [22, 23] or conformational changes [24], we have used it here to read out changes in the KOP-eGFP immediate environment that are caused by treatment of PC12/KOP-eGFP cells with EtOH or NTX. Fluorescence correlation spectroscopy (FCS) was used to measure the lateral translational diffusion rate and the cell surface density, i.e., concentration of molecules of interest, such as KOP-eGFP, fluorescently labeled NTX and lipid probes, and read out how they are affected by EtOH and NTX [20, 25, 26]. In addition, FCS also measures molecular brightness, providing information on homodimerization/oligomerization of KOP-eGFP. We have also characterized EtOH effects on NTX-KOP interactions using fluorescently labeled NTX (fNTX; Fig. S1) [27]. Finally, Ca^2+^ imaging was used to characterize the effects of the investigated compounds on KOP-mediated signaling.

## Materials and methods

### Chemical reagents

Ethanol (EtOH, purity ≥99.5%) and NTX were purchased from VWR and Tocris, respectively. Methyl-β-cyclodextrin (mβCD) and Nalfurafine (NFF) were purchased from Sigma-Aldrich. Dynorphin A (1–17) peptide (DynA: YGGFLRRIRPKLKWDNQ; >99.5% purity) was purchased from BIOMATIK. The NTX enantiomer (+)-NTX was kindly provided by Dr. Kenner C. Rice [28]. The fluorescent NTX derivative with Alexa Fluor 633 (fNTX) was synthesized as detailed in the Supplementary Information (Fig. S1). The KOP-selective antagonist, LY2444296 was supplied by Eli Lilly, and the KOP-selective antagonist JDTic [29] was purchased from APExBIO. All chemical compounds except for EtOH, DynA and (+)-NTX were suspended in dimethyl-sulfoxide (DMSO). DynA and (+)-NTX aqueous solutions were freshly prepared for each experiment. 1,2-dioleoyl-sn-glycero-3-phosphoethanolamine (DOPE) was conjugated with Abberior Star Red with a polyethylene glycol (PEG) linker (ASR-DOPE) [30]. MemGlow Nail Red 12S (NR12S) for lipid fluidity studies was purchased from Cytoskeleton, Inc. All chemicals were *p.a*. grade and were used without further purification, unless specifically described. Ultrapure water, resistivity 18.2 MΩ·cm at 25 °C (Millipore Milli-Q lab water system) was used throughout.

### Cell culture

PC12 cells (American Type Culture Collection), PC12/eGFP cells transiently expressing eGFP, and PC12 cells stably expressing human KOP fused with the enhanced green fluorescent protein (PC12/KOP-eGFP) [31] were maintained in a humidified atmosphere containing 5% CO_2_ at 37 °C in RPMI1640 medium (Gibco) supplemented with 10% horse serum (Gibco), 5% fetal bovine serum (Gibco) and 1% penicillin-streptomycin (10,000 U/mL, Gibco). For fluorescence measurements, the cells were seeded in Lab-Tek 8-well chambered coverglass (Thermo Fisher Scientific) with 4.0 × 10^4^ cells/well.

For FLIM/FRAP measurements, PC12/KOP-eGFP cells were pre-treated at 37 °C with the antagonists NTX or (+)-NTX for 30 min, or LY2444296 for 15 min; or the agonists DynA or NFF for 30 min, and then treated with EtOH + antagonists/agonist for 1 h. For treatments with EtOH alone, the cells were pre-treated with vehicle for 30 min, then with EtOH for 1 h.

For cholesterol depletion studies, the cells were treated for 3 h with 2.5 mM mβCD in serum free medium at 37 °C [32]. Antagonists, agonists, mβCD and EtOH were diluted with the FluoroBrite RPMI1640 (Gibco).

For Fluorescence Correlation Spectroscopy (FCS) measurements of lipid fluidity or KOP-eGFP dynamics, PC12/KOP-eGFP cells were treated with the antagonists/EtOH or DynA as described above. The cells were further stained with ASR-DOPE for 5 min. To obtain eGFP brightness, PC12/eGFP cells were used. PC12/eGFP cells were generated by transfecting PC12 cells with 100 ng of the plasmid encoding eGFP, peGFP-N1, using 0.2 µL of lipofectamine 2000 (Thermo Fisher Scientific). After the transfection, PC12/eGFP cells were cultured for 24 h and then subjected to FCS measurements.

For Ca^2+^ imaging, PC12/KOP-eGFP cells were stained with 10 µM Fura Red in non-serum FluoroBrite RPMI1640 with 0.1% Pluronic F-127 (Invitrogen) for 3 h. Antagonists, DynA and EtOH were diluted with Dulbecco’s Phosphate Buffered Saline supplemented with 2.2 mM CaCl_2_, and 3.5 mM KCl. PC12/KOP-eGFP cells were treated with antagonists/EtOH or DynA/NTX as described above.

For total internal reflection fluorescence microscopy-integrated FCS (TIR-FCS), we specifically used another cell line, the adhered human osteosarcoma cell line, U2OS (ATCC). U2OS cells were maintained in a humidified atmosphere containing 5% CO_2_ at 37 °C in McCoy’s 5 A modified medium (Gibco) supplemented with 10% FBS. One day before transfection, the U2OS cells were seeded on the 8-well chambered coverglass. U2OS cells on the chambered coverglass were transfected with 100 ng of plasmid DNA encoding human KOP-eGFP in N1 vector (pKOP-eGFP-N1) and ViaFect (Promega). At 24 h after the transfection, the medium was replaced with a phenol-red free medium, Opti-MEM (Gibco), for TIR-FCS experiments.

### Microscopic techniques and corresponding data analyses

Detailed description of the instrumentation for FCS, TIR-FCS and FLIM, optical settings, data acquisition and analysis is provided in the Supplementary Information. Briefly, confocal laser scanning microscopy (CLSM) imaging and conventional, single-point FCS measurements were performed using the LSM880 (Carl Zeiss) microscope system. TIR-FCS measurements [33–35] were performed using the Nikon Eclipse TE2000-E inverted microscope with a TIRF unit. FLIM was performed using our home-built scanning-free confocal microscope based on massively parallel fluorescence correlation spectroscopy (mpFCS) [22].

## Results

### Live PC12/ KOP-eGFP cells are functional

To ascertain that KOP-eGFP is functional, PC12/KOP-eGFP cells were treated with DynA (Fig. S2). CLSM imaging showed that in untreated PC12/KOP-eGFP cells, KOP-eGFP is largely localized in the plasma membrane, with some trafficking vesicles binding visible in the cytoplasm (Fig. S2, upper left corner). Massive KOP-eGFP internalization is observed after 30 min treatment with 100 nM DynA (Fig. S2, upper right corner). Treatment with NTX does not alter KOP-eGFP distribution (Fig. S2, lower left corner), but efficiently blocks DynA-induced internalization (Fig. S2, lower right corner).

We have also characterized using FCS the mobility of DynA-activated KOP-eGFP in the plasma membrane (Fig. S3). The observed slow lateral diffusion and its further lowering following treatment with DynA is indicative of KOP-eGFP localization in nanoscale clusters, a common feature of plasma membrane receptors [10–12].

**Fig. 1:**
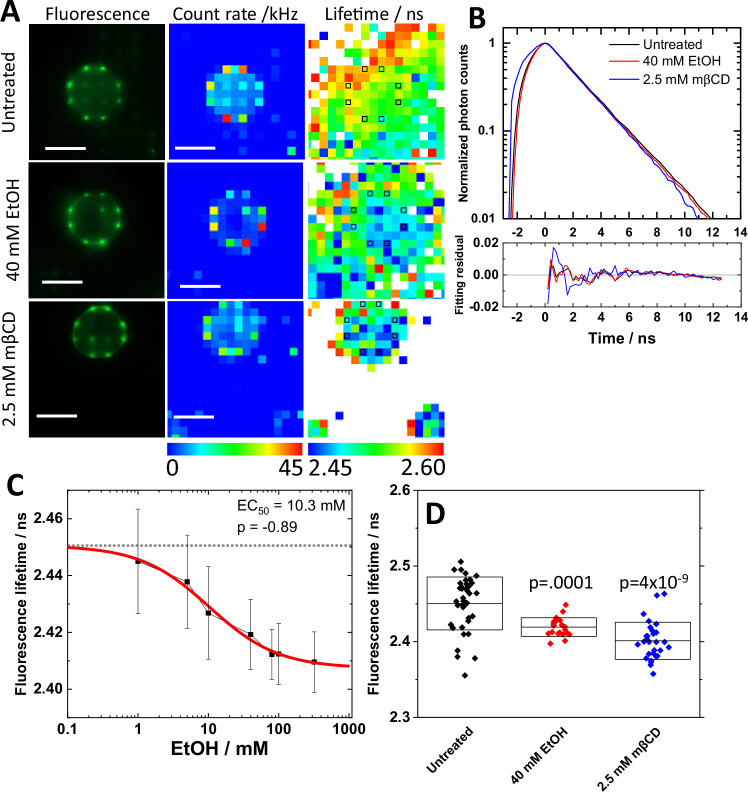
eGFP fluorescence lifetime (FL) measured in live PC12/KOP-eGFP cells changes in response to ethanol treatment in a dose-dependent way. **A** Fluorescence images/photon counts map acquired using a high-resolution (2 mega pixel) CMOS camera (left column) and a 2D spc3 SPAD camera (middle column). FL map (right column) generated by fitting analysis of FLIM curves shown in **B**. Black squares indicate plasma membrane positions assigned by photon count map. Scale bar: 10 µm. **B** FLIM curves and corresponding fit residuals. Black: Untreated. Red: 40 mM EtOH. Blue: 2.5 mM mβCD. **C** Dose-response curve showing the magnitude of change in eGFP FL as a function of EtOH concentration. Best fit of dose-response curve determined 10.3 mM and −0.89 as EC_50_ value and allosteric factor (p), respectively. **D** eGFP FL, given as average ± standard deviation, under the treatment with 40 mM EtOH and 2.5 mM mβCD. Statistical analysis was performed using the two-tailed Student’s t-test against untreated.

FLIM measurements (Fig. 1A, B), performed using our in-house instrument [22, 36], showed that treatment with 100 nM DynA significantly decreases eGFP FL (Fig. S4). Importantly, both NTX and the KOP-selective antagonist JDTic blocked this effect, suggesting that DynA binds to KOP-eGFP in the plasma membrane and that the observed change in eGFP FL is due to KOP-eGFP-mediated effects.

Finally, Ca^2+^ imaging was performed following extracellular K^+^-induced membrane depolarization of cultured PC12/KOP-eGFP cells (Fig. S5). Ca^2+^ imaging showed that treatment with 100 nM DynA decreased the Ca^2+^ influx induced by the abrupt increase in extracellular K^+^, in line with the notion that DynA-activated KOPs released G-protein subunits, inhibiting the Ca^2+^ ion channel *via* the released G_βγ_ subunits. 200 nM NTX blocked the 100 nM DynA-induced change in Ca^2+^ influx (Fig. S5).

Taken together, these data indicate that KOP-eGFP in PC12/KOP-eGFP cells is functional and that changes in eGFP FL are reliable indicators of KOP-eGFP activation status.

### Ethanol induces dose-dependent changes in eGFP fluorescence lifetime

Ethanol is known to affect membrane lipid structures [37–39] and the lateral organization of KOP in the plasma membrane [8]. To determine whether these ethanol-induced changes can also lead to KOP activation, we have performed FLIM on PC12/KOP-eGFP (Fig. 1). As can be seen, KOP-eGFP fluorescence was localized in the plasma membrane in untreated PC12/KOP-eGFP cells and in cells treated with 40 mM EtOH (Fig. 1A, top and middle rows), showing that EtOH treatment does not induce receptor internalization as observed with the DynA agonist (Fig. S2). Furthermore, FLIM curves recorded in the plasma membrane showed measurable differences in eGFP FL in response to different treatments (Fig. 1B). For ethanol treatment, the change in eGFP FL depended on the ethanol concentration, showing a dose-dependent change with a half-maximal effective concentration (EC_50_) of 10.3 mM (Fig. 1C). Since dynamic properties of plasma membrane lipids and the lateral organization and size of nanoscale clusters harboring KOP-eGFP are influenced by cholesterol-enriched membrane domains [32], FLIM measurements were also performed under cholesterol depletion by 2.5 mM mβCD. As can be seen, eGFP FL significantly decreased upon cholesterol depletion from the plasma membrane (Fig. 1D), suggesting that EtOH affects dose-dependently the KOP-surrounding membrane environment including a change in the cholesterol-enriched plasma membrane domains.

### EtOH affects NTX binding to KOP

**Fig. 2:**
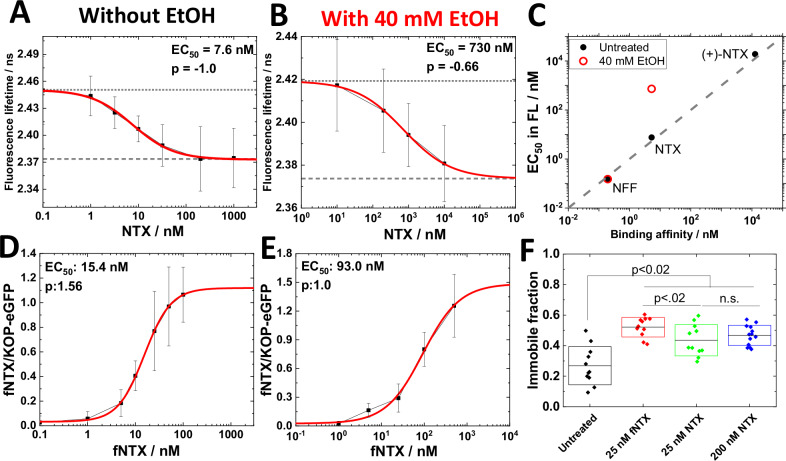
Effect of ethanol on NTX interactions with KOP-eGFP. **A**, **B** Dose-response curve showing the magnitude of change in eGFP FL as a function of the concentration of NTX recorded in untreated cells (**A**) and cells treated with 40 mM EtOH (**B**). Best fit of dose-response curves yielded EC_50_ and allosteric factor (p) values of 7.6 nM and −1.0 (**A**) and 730 nM and −0.66 (**B**), respectively. **C** Correlation between the EC_50_ value measured using FLIM with literature binding affinity values of agonist/antagonist to KOP: Nalfurafine (NFF), naltrexone (NTX) and (+)-NTX. Plotted binding affinity values are taken from [40, 42, 43]. Binding affinity of (+)-NTX to KOP was estimated from binding affinity of (+)-NTX to MOP and the ratio of binding affinities of NTX to MOP and KOP. Black: Vehicle. Red: 40 mM EtOH. **D**, **E** Dose-response curve showing the magnitude of change in the fluorescence intensity ratio in the plasm membrane (*F*_NTX_/*F*_KOP-eGFP_) as a function of the concentration of fNTX recorded in untreated cells (**D**) and cells treated with 40 mM EtOH (**E**). Best fit of the dose-response curves yielded EC_50_ and allosteric factor (p) values of 15.4 nM and 1.56 (**D**) and 93.0 and 1.0 (**E**), respectively. **F** Changes in the immobile fraction of KOP-eGFP in the plasma membrane assessed by FRAP. Black: Untreated. Red: treated with 25 nM fNTX. Green: treated with 25 nM NTX; Blue: treated with 200 nM NTX. Statistical analysis was performed using the two-tailed Student’s *t* test.

We have previously observed that NTX enhances the formation of larger nanoscale clusters harboring KOP [8], blocks DynA binding (Figs. S2 and S4) and DynA-induced KOP-eGFP-mediated actions (Fig. S5). To assess whether EtOH affects NTX binding and actions in the plasma membrane, FLIM was performed on PC12/KOP-eGFP cells treated with NTX or NTX+EtOH (Fig. 2). Our data showed that NTX decreased eGFP FL in a dose-dependent manner, with an EC_50_ value 7.6 nM (Fig. 2A). To confirm that these changes in eGFP FL are KOP-eGFP-mediated, we also assessed the effects of two related compounds: the inactive optical isomer of NTX, (+)-NTX [40] (Fig. S6) and the KOP-selective agonist nalfurafine (NFF) [41] (Fig. S7). Each compound caused changes in eGFP FL, with an EC_50_ value of 19 µM for (+)-NTX (Fig. S6A) and 0.15 nM for NFF (Fig. S7B). Interestingly, the binding affinity measured using FLIM agreed well with the binding affinity of these compounds to KOP determined in other studies [40, 42, 43] (Fig. 2C). Given that PC12 cells endogenously express DynA, albeit at very lower levels [44], the good agreement between EC_50_ values measured by FLIM with literature findings suggests that endogenously expressed DynA exerts negligible competing binding effects. Finally, in line with what is expected, treatment with 200 nM NTX efficiently blocked 1 nM NFF binding (Fig. S7C), further corroborating our interpretation of the FLIM data.

Importantly, under treatment with 40 mM EtOH the dose-response curve for NTX shifted to higher NTX concentrations, as reflected by a change in the EC_50_ value from 7.6 nM, measured in untreated cells, to 730 nM, measured in cells treated with 40 mM EtOH (Fig. 2B). 40 mM EtOH disrupted the dose-response activity of (+)-NTX (Fig. S6B, C). In contrast, 40 mM EtOH did not change the EC_50_ value of NFF (Fig. S7D).

To further examine whether the high EC_50_ value for NTX binding in the presence of 40 mM EtOH measured by FLIM, ${{EC}}_{50,{NTX}+40{mMEtOH}}^{{FLIM}}=730{\rm{nM}}$, is indeed due to EtOH-induced lowering in NTX binding affinity, fNTX was synthesized and its binding to PC12/KOP-eGFP cells was characterized by CLSM (Fig. S8). As can be seen, fNTX binding to KOP-eGFP in live PC12/KOP-eGFP cells is significantly reduced in the presence of 40 mM EtOH and was efficiently blocked by a large excess of non-labeled NTX or by the KOP-selective antagonist LY2444296 (Fig. S8). This not only verified the observations by FLIM that EtOH lowers NTX binding affinity to KOP, but also confirmed that fNTX specifically binds to KOP-eGFP. Dose-response curves showing changes in the fluorescence intensity ratio (*F*_fNTX_/*F*_KOP-eGFP_) as a function of fNTX concentration showed EC_50_ values of 15.4 nM for fNTX treatment, and 93 nM for fNTX+40 mM EtOH treatment (Fig. 2D, E). NTX binding affinity to KOP was lower under combined treatment with 40 mM EtOH (Fig. S8B, black *vs* red line), in agreement with our FLIM data (Fig. 2A–C).

FRAP analysis (Fig. 2F) suggests that both NTX and fNTX increase the immobile fraction of KOP-eGFP in the plasma membrane, presumably by sorting the KOP-eGFP receptors to KOP-harboring clusters. This data confirmed that fNTX retained functionality similar to that of NTX.

### NTX reduces EtOH effect on lipid dynamics

**Fig. 3:**
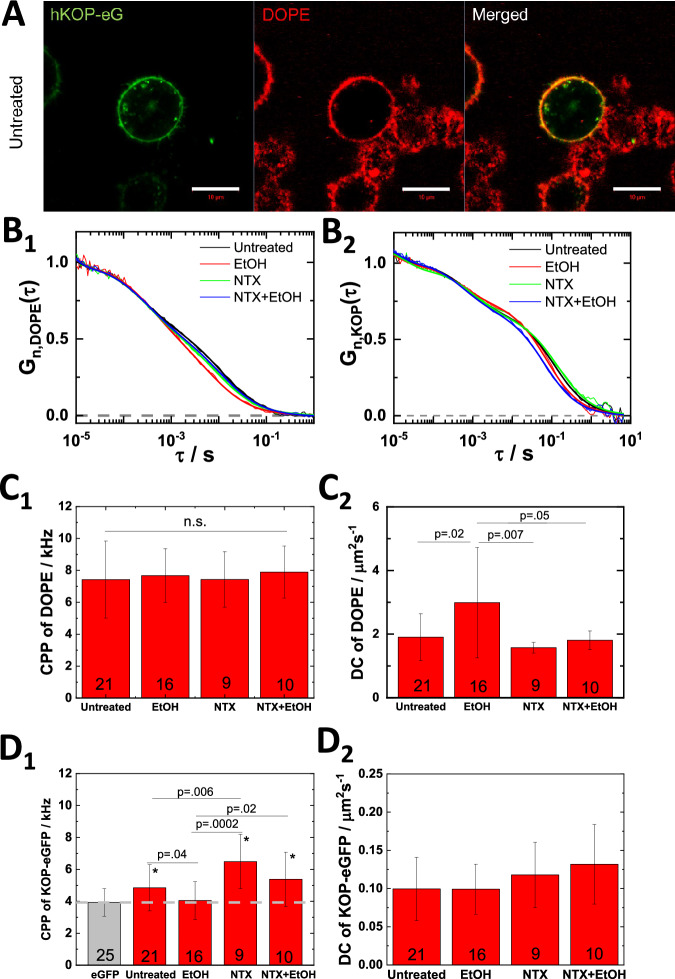
NTX wards off EtOH-induced effects on the lateral lipid dynamics and KOP oligomerization. **A** Confocal fluorescence microscopy images of untreated PC12/KOP-eGFP cells (green) stained with ASR-DOPE (DOPE, red). Scale bar: 10 µm. (**B**_**1**_**, B**_**2**_) Normalized autocorrelation curves (ACCs) reflecting ASR-DOPE (**B**_**1**_) and KOP-eGFP (**B**_**2**_) dynamics in the plasma membrane of untreated and treated PC12/KOP-eGFP cells. Black: untreated. Red: treated with 40 mM EtOH. Green: treated with 200 nM NTX; Blue: treated with 200 nM NTX + 40 mM EtOH. The ACCs are normalized to the same amplitude, *τ*_D_ = 1 at *τ* = 10 µs. **C**_**1**_**, C**_**2**_ Fitting results from ASR-DOPE. Counts per particle (CPP) (**C**_**1**_) and diffusion coefficient (DC) (**C**_**2**_) of membrane-bound component of ASR-DOPE. **D**_**1**_**, D**_**2**_ Fitting results from KOP-eGFP and eGFP (gray). CPP (**D**_**1**_) and DC of slow component (**D**_**2**_) of KOP-eGFP. eGFP was used as brightness standard for the monomeric form of KOP-eGFP. The number of measured single cells is shown at the bottom of bars in **C**, **D**. Statistical analysis was performed using the two-tailed Student’s *t* test. Asterisk (*) indicates significance, p < 0.01, for comparisons against eGFP.

Taken together, our data presented above suggests that EtOH affects the KOP-eGFP surrounding lipid environment, including a change in the lateral organization and diffusion of cholesterol-enriched membrane domain (Fig. 1). To further investigate EtOH effects on the lipid environment in the plasma membrane, we selected the lipid marker Abberior Star Red-labeled DOPE (ASR-DOPE) probe. ASR-DOPE clearly stained the plasma membrane and colocalized with KOP-eGFP in untreated PC12/KOP-eGFP cells (Fig. 3A) and EtOH-treated PC12/KOP-eGFP cells (Fig. S9). Fluorescence Correlation Spectroscopy (FCS) measurements were performed on both, ASR-DOPE (Fig. 3B_1_, C_1_, C_2_) and KOP-eGFP (Fig. 3B_2_, D_1_, D_2_). FCS measurements on ASR-DOPE yielded autocorrelation curves (Fig. 3B_1_) with two distinct decay times that reflect ASR-DOPE diffusion in the cell culture medium (short decay time) and in the plasma membrane (long decay time). The counts per particle (CPP), i.e., brightness of the ASR-DOPE probe was not significantly changed under any treatment tested (Fig. 3C_1_); whereas ASR-DOPE diffusion in the plasma membrane was significantly increased in PC12/KOP-eGFP cells treated with 40 mM EtOH (Fig. 3C_2_). Pretreatment with 200 nM NTX warded off the EtOH-induced effects (Fig. 3C_2_). To address whether this is an NTX-specific or general effect of KOP antagonists on EtOH-modulated effects on lipid dynamics, we also characterized the effects of treatment with (+)-NTX and the KOP-selective antagonist LY2444296 (LY) [45, 46]. In line with the results by FLIM (Fig. S6), we observed that treatment with 200 nM (+)-NTX did not ward off the EtOH-induced effects on lipid dynamics. Of note, treatment with (+)-NTX at a very large, 500-fold excess compared to the effective NTX concentration, 100 µM (+)-NTX, shows NTX-like effect on the lipid dynamics (Fig. S10). Warding off EtOH-induced effects were not observed for treatment with 100 nM LY2444296 (Fig. S11). Taken together, these data suggest that NTX effects are not KOP-mediated only. Finally, we confirmed the effects of EtOH and NTX by examining in wild type PC12 cells their effects on ASR-DOPE diffusion (Fig. S12) and plasma membrane fluidity using General Polarization (GP) analysis (Fig. S13). Our data suggest that EtOH enhances lipid fluidity in the plasma membrane, as reflected by increased ASR-DOPE diffusion and a statistically significant decrease in plasma membrane GP, and that NTX wards off these EtOH-induced effects.

### NTX wards off EtOH-induced decrease in KOP homodimer population in the plasma membrane

FCS analysis showed that the apparent average brightness of KOP-eGFP, as reflected by counts *per* particle (CPP), was significantly higher than that of eGFP, which is a good proxy for monomeric KOP-eGFP, but less than twice this value (Fig. 3D_1_). This is indicative of partial KOP-eGFP homodimerization, which is common in GPCRs [47–49]. EtOH significantly reduced the CPP of KOP-eGFP, lowering it down to the level of eGFP (Fig. 3D_1_). This suggests that in EtOH-treated PC12/KOP-eGFP cells KOP-eGFP homodimers dissociate to the monomeric state. In contrast, NTX increased the CPP (Fig. 3D_1_), presumably enhancing KOP-eGFP homodimerization and warded off the EtOH-induced dissociation of KOP-eGFP homodimers. Interestingly, this effect was also observed in cells treated with (+)-NTX, unless it was used in a very large excess (100 µM), but not at concentrations that are relevant for NTX actions (200 nM; Fig. S10D). Unlike NTX, the KOP-selective antagonist LY2444296 did not interfere with EtOH-induced dissociation of KOP-eGFP dimers (Fig. S11C) and neither affected the lateral diffusion of lipids in the plasma membrane (Fig. S11B), nor KOP-eGFP dimerization, as reflected by CPP values that were not statistically significantly different from the value measured in untreated cells (Fig. S11C).

To further characterize KOP-eGFP lateral organization, we performed TIR-FCS [50, 51], using diffusion law analysis to assess KOP-eGFP confinement in membrane domains (Fig. S14). The diffusion law analysis examines the relationship between KOP-eGFP diffusion time (τ_D_) as a function of the observation area (A_eff_). For free diffusion, the diffusion time linearly increases as the observation area increases and the intercept of the linear regression is 0. For KOP-eGFP, a positive intercept is observed, which is an indication that KOP-eGFP is confined in domains (Fig. S14). In PC12/KOP-eGFP cells treated with 40 mM EtOH, the intercept of the linear regression τ_D_ = *f*(A_eff_) was significantly lower (Fig. S14D), suggesting that EtOH reduces the fraction of KOP-eGFP confined in domains. While conventional, single-point FCS could not observe treatment-related differences in KOP-eGFP diffusion coefficient (DC) in PC12/KOP-eGFP cells (Fig. 3D_2_), a significant difference was observed by TIR-FCS in U2OS cells transiently expressing KOP-eGFP (Fig. S14C). We attribute this difference to differences in sensitivity between TIR-FCS and conventional FCS, rather than to differences in cell type. TIR-FCS uses total internal reflection to illuminate the sample, thus confining the excitation light into a thin (100–200 nm) region near the basal plasma membrane of the cell. This significant decrease in the observation volume reduces significantly background fluorescence and leads to a higher signal-to-noise ratio (SNR) and therefore greater sensitivity of TIR-FCS as compared to conventional FCS.

Since FCS can only observe the mobile pool of KOP-eGFP molecules in the plasma membrane and cannot give any information about the immobile fraction, we resorted to FRAP to assess to what extent the immobile pool of KOP-eGFP is affected by the investigated treatments. Our data show that EtOH reduced the immobile fraction of KOP-eGFP, whereas NTX increased it and warded off EtOH-induced changes (Fig. S15). In line with our published observation of KOP-harboring nanoscale domains [8], we propose that KOP-eGFP sequestered into domains constitute the immobile receptor pool; that EtOH redistributes KOP-eGFP between the domains and the surrounding lipid bilayer, shifting the equilibrium towards the mobile fraction. In contrast, NTX shifts the equilibrium towards KOP-eGFP sequestration in protein- and lipid-enriched plasma membrane domains, thereby counteracting EtOH-induced KOP-eGFP reorganization. In FRAP experiments, this is observed as EtOH-/NTX-induced rescinding/retention of the immobile KOP-eGFP fraction.

Taken together with FCS brightness analysis, our data suggest that EtOH and NTX affect KOP-eGFP association with surrounding lipid environment, modulating nanoscale cluster formation and the number of KOP-eGFP molecules in the cluster, followed by an EtOH-induced decrease/NTX-induced increase of KOP-eGFP homodimers.

### EtOH and NTX modulate Ca^2+^ signaling *via* KOP-dependent and independent pathways

**Fig. 4:**
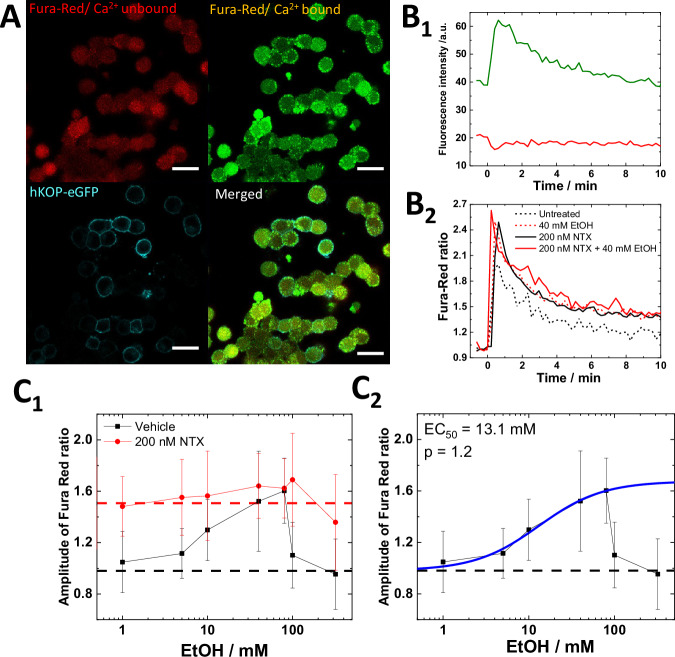
EtOH and NTX modulate Ca^2+^ signaling via KOP-dependent and -independent pathways. **A** Confocal fluorescence microscopy images of untreated PC12/KOP-eGFP stained with Fura Red. Red: Ca^2+^-unbound Fura Red. Green: Ca^2+^-bound Fura Red. Cyan: KOP-eGFP. Scale bar: 20 µm. **B**_**1**_ Changes in Fura Red fluorescence intensity over time during K^+^ depolarization. Green: Ca^2+^-bound Fura Red, Red: Ca^2+^-unbound Fura Red. **B**_**2**_ Fura Red ratio normalized before the K^+^ stimulation. Black dashed line: Untreated. Red dashed line: 40 mM EtOH. Black solid line: 200 nM NTX. Red solid line: 200 nM NTX + 40 mM EtOH. **C**_**1**_ The magnitude of change in the amplitude of Fura Red ratio as a function of the concentration of EtOH. Black: vehicle + EtOH, Red: 200 nM NTX + EtOH. Dashed lines reflect mean values for vehicle (black) and for 200 nM NTX-treated cells (red). **C**_**2**_ Best fit of dose-response curve (blue) as a function of EtOH concentration yielded EC_50_ and allosteric factor (p) values of 13.1 mM and 1.2, respectively.

To assess how EtOH and NTX affect the function of KOP-eGFP, we performed Ca^2+^ imaging in PC12/KOP-eGFP cells using the Ca^2+^-sensitive fluorescent dye Fura Red (Fig. 4A). After K^+^ depolarization, fluorescence intensity of Ca^2+^-bound Fura Red dramatically increased while fluorescence intensity of Ca^2+^-unbound Fura Red decreased (Fig. 4B_1_). The Fura Red ratio was calculated as the ratio of Ca^2+^-bound intensity and Ca^2+^-unbound intensity, which is reflecting on the intercellular Ca^2+^ ion concentration (Fig. 4B_2_). We assessed changes in the amplitude of Fura Red ratio generated by K^+^ depolarization as a proxy for Ca^2+^ influx induced by the depolarization. EtOH-treated PC12/KOP-eGFP cells showed a gradual, dose-dependent increase of the amplitude of Fura Red ratio for EtOH concentrations of up to 80 mM EtOH. For EtOH concentrations ≥ 100 mM, a sudden drop was observed (Fig. 4C_1_). The EC_50_ value was determined to be 13.1 mM EtOH (Fig. 4C_2_), in good agreement with the EC_50_ value determined by FLIM (10.3 mM) (Fig. 1C). Cholesterol depletion caused similar changes of the amplitude of Fura Red ratio (Fig. S16), suggesting that EtOH induces Ca^2+^ influx by modulating the lipid surroundings of KOP-eGFP, including formation/deformation of cholesterol-enriched KOP-eGFP harboring plasma membrane domains. To ascertain if this is a KOP-mediated pathway or not, Ca^2+^ imaging was performed in untransfected, wild-type PC12 cells, showing a higher Fura Red ratio in untreated PC12 cells (1.3 ± 0.2; Fig. S17, black dashed line) compared to untreated PC12/KOP-eGFP cells (1.0 ± 0.2; Fig. 4C_1_, black dashed line) and a gradual decrease of the amplitude of Fura Red ratio, rather than its enhancement (Fig. S17). This is in good agreement with previous studies showing that the L-type channel is inhibited and the non-L-type channel is partially inhibited by EtOH [52]. Furthermore, EtOH did not affect the baseline of Fura Red ratio before K^+^ depolarization (Fig. S18), suggesting that pharmacologically relevant concentration of EtOH (~10 mM) does not affect the intercellular Ca^2+^ ion concentration and also does not induce endoplasmic reticulum (ER) stress, as reported in pancreatic acinar cells using extremely high EtOH concentration [53]. Considering the lower effect of endogenous DynA in PC12 cells, which is described in the section “EtOH affects NTX binding to KOP”, this suggests that KOP activated by thermal fluctuations, which may be localized in cholesterol-enriched membrane domains, partially exists even in the absence of agonists, inhibiting Ca^2+^ influx at the basal level and that EtOH treatment affects components in the plasma membrane, enhancing Ca^2+^ influx through a KOP-mediated pathway.

To clarify the impact of NTX on Ca^2+^ influx, we pre-treated PC12/KOP-eGFP cells and wild type PC12 cells with 200 nM NTX. NTX-treated PC12/KOP-eGFP cells showed higher amplitude of Fura Red ratio (Fig. 4C_1_), which is opposite to the change observed in DynA-treated cells (Fig. S5). Likely, this suggests that NTX downregulates the inhibition of the Ca^2+^ ion channels *via* KOP activation and the subsequent release of G_βγ_ subunits. Interestingly, a constant amplitude of the Fura Red ratio was observed even for treatments with EtOH concentrations >100 mM (Fig. 4C_1_). The constant amplitude of Fura Red ratio was also confirmed in wild type cells (Fig. S17). This may suggest that EtOH-induced disruption of the Ca^2+^ influx for EtOH concentrations >100 mM and the gradual decrease of Ca^2+^ influx observed in wild type PC12 cells are due to changes in the plasma membrane, including membrane domains, and that NTX blocked this EtOH-induced effect, thereby retaining constant amplitude of Fura Red ratio across all EtOH concentrations tested.

In summary, our data suggest that NTX actions in live PC12/KOP-eGFP cells are complex; occurring through two principal pathways: 1) direct binding to KOP-eGFP and 2) KOP-unmediated modulation of cholesterol-enriched membrane domains, which is in good agreement with our FCS data (Fig. 3) and previous work [8].

To further characterize the contribution of these two pathways, effects of NTX on Ca^2+^ influx were tested using (+)-NTX (Fig. S19) and LY2444296 (Fig. S20). 200 nM (+)-NTX clearly showed no effect on Ca^2+^ influx (Fig. S19, red), while 100 µM (+)-NTX slightly increased Ca^2+^ influx without EtOH treatment, following a similar pattern under co-treatment with EtOH (Fig. S19, blue). LY2444296 (100 nM) was found to enhance Ca^2+^ influx, a direct KOP antagonistic effect achieved *via* binding to KOP-eGFP (Fig. S20), but did not show any KOP-unmediated effect (Fig. S11).

Taken together, our FLIM data (Figs. 1 and 2) and FCS data (Fig. 3), suggest that EtOH modulates Ca^2+^ influx *via* changes of membrane environment, in particular deformation of cholesterol-enriched membrane domains. NTX does not only show the KOP-mediated antagonistic effects on Ca^2+^ influx, but also KOP-unmediated effects on lipid dynamics in the receptor surrounding lipid environment, which is related to KOP-harboring nanoscale cluster formation.

## Discussion

Alcohol abuse and dependence remain the most significant substance abuse problems worldwide and in the US alone more than 140,000 people are dying from alcohol-related causes annually [54]. While there is an ongoing debate whether psychotherapy or medication have superiority for treatment, it is a fact that the majority of individuals with AUD receive no treatment at all.

Earlier studies of medication in AUD have been subjected to a meta-analysis, showing the NTX and acamprosate are superior to placebo; NTX is particularly effective in heavy drinking and prevention of recurrence [55]. A more recent survey supports the efficacy of NTX. Moreover, effectiveness of extended-release NTX medication was recently demonstrated [56]. However, NTX-based prescriptions were primarily given to higher income males with private insurance, leaving women and minorities without such intervention [57]. A certain rise in AUD have been recorded during the covid-19 pandemic [58], prompting an editorial advocating higher rate of NTX prescriptions [1].

Behavioral effects mediated by KOP differ markedly from those of the other opioid receptors, MOP and the delta-opioid receptor (DOP). Kappa-agonists are not self-injected and clinical use is compromised by psychotomimetic side effects. It has even been proposed that the overt euphorigenic effects of MOP and DOP pathways are related to positive reinforcement whereas effects on KOP are balancing and related to the negative reinforcement (craving) [59]; both effects have been related to AUD.

NTX has found a therapeutic niche for the treatment of AUD and is one of the very few medications that can be prescribed for this indication. One characteristic of AUD in humans is that dependent subjects will consume alcohol to relieve or avoid withdrawal symptoms. Similarly, in preclinical studies, alcohol postdependent rats exhibit an alcohol dependence syndrome that is characterized by both somatic and motivational withdrawal symptoms that usually begin after 6 to 8 h of abstinence and engage in excessive drinking when alcohol is made available again. Using a rat model of alcohol dependence (i.e, chronic intermittent alcohol vapor exposure) we showed that NTX decreased alcohol intake in nondependent rats, regardless of sex and abstinence time point [60]. In postdependent rats, NTX significantly decreased the exaggerated alcohol intake only at a delayed abstinence time point (i.e., 6 weeks) in males, whereas it similarly reduced alcohol drinking in females at 8 h, 2 weeks, and 6 weeks abstinence time points. These findings further support targeting the endogenous opioid system to prevent excessive drinking that is characteristic of AUD, even after long periods of abstinence and further suggest that alcohol dependence causes neuroadaptation [60].

The access of a fluorescent derivative of NTX was a priority in the study. While fluorescent NTX derivatives have been described before [27], the strategy here was to extend the separation of the fluorescent marker to NTX by a longer linker, considering the X-ray analysis data showing that the JDTic, a KOP antagonist, binds in a deep pocket [14] and NMR analysis of the KOP/dynorphin interaction [15].

NTX has been reported to have a lower affinity for KOP as compared to MOP and may therefore not be considered a KOP antagonist. However, under our conditions, affinity is strong and more in line with previous studies using competition assays in transfected cell cultures that identified approximately equal affinity of NTX for KOP and MOP [61]. Constitutive activity and inverse agonism were also observed, which increased after agonist pretreatment [61]. To approach the effects of alcohol on both MOP and KOP at a molecular level, we introduced high-resolution molecular imaging with FCS to follow the dynamics in cell culture of MOP and KOP labeled with fluorescent tags. The addition of pharmacologically relevant concentrations of EtOH influenced their lateral movements in the plasma membrane [62]. Significantly, EtOH-induced effects showed differences between MOP and KOP, with higher presence of MOP in the membrane, whereas KOP presence declined. Differences related to EtOH-induced effects were also observed with super-resolution microscopy [8].

In our studies, we have also used high-resolution technologies to investigate the effects of EtOH on both MOP and KOP in cell culture. As expected, NTX blocks the activation of MOP. At the ultrastructure, nanoscale level, EtOH affects the distribution of both MOP and KOP (induces the formation of smaller and less occupied MOP and KOP nanodomains) [8]. These studies also revealed that NTX induces formation of larger and more occupied KOP nanodomains and that NTX pretreatment has protective effects against EtOH-induced changes in nano-organization of both receptors [8].

Another approach to the specificity of the studies effects is the use of stereoisomers. The (+) isomer of NTX ((+)-NTX) was available to us. This isomer has its own pharmacologic profile and shows equipotent binding for MOP and the toll-like receptor TR4, and interacts with opioid (morphine) analgesia [63, 64] as well as drug reward [65]. The results are clear, the (+) isomer is much less active demonstrating that the NTX effects we observe are mediated by interaction with KOP and not due to off-target effects (Fig. 2).

**Fig. 5:**
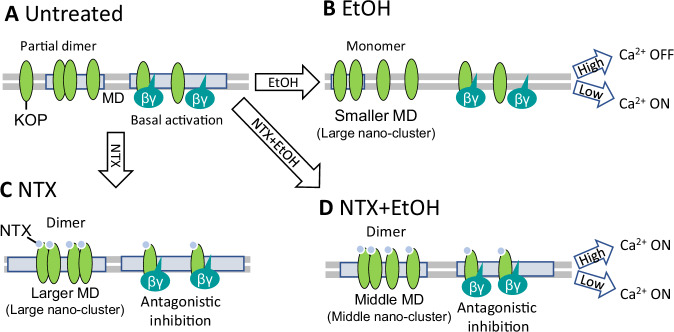
Model of NTX and ethanol effects on KOP lateral organization and interactions with lipids. **A** In untreated cells, KOP largely diffuses freely in the lipid bilayer, with a small fraction that localizes in cholesterol-enriched membrane domains (MDs) and forms homodimers. KOPs that localize in MD can be activated by thermal fluctuations, even without the presence of an externally introduced agonist. Through this basal activation of KOP, KOP-mediated inhibition of Ca^2+^ channel can take place. **B** Under treatment with EtOH, deformation of MD is taking place, KOP dimers dissociate, forming monomers that freely diffuse in the lipid bilayer. This, in turn leads to reduction in the KOP-mediated inhibition of the Ca^2+^ channel, and the Ca^2+^ channel is in the ON-state under lower EtOH concentrations (0 mM–80 mM). Under high EtOH concentration (>100 mM), plasma membrane deformation is excessive and the Ca^2+^ channel may be generally inhibited. **C** Under the treatment with NTX, larger nanoscale KOP clusters form (based on evidence from [8]). NTX-bound KOP forms predominantly homodimers in larger MDs. The Ca^2+^ channel is released from the inhibiting action of KOP by NTX binding. **D** EtOH-modulated lipid dynamics is suppressed by NTX. Middle-sized nano-clusters are being formed. NTX also exerts its antagonist activity at KOP, thus the Ca^2+^ channel is released from KOP-activated-inhibition.

Based on our data, we have developed a model to describe EtOH, and the direct and indirect actions of naltrexone (NTX) on KOP function (Fig. 5). In untreated cells under normal culture conditions, KOPs exist partly as monomers and partly as homodimers. They are distributed between the lipid bilayer and nanoscale clusters within the plasma membrane. Under these conditions, thermal fluctuations can activate the fraction of KOPs localized in clusters, resulting in a basal level of KOP activity even in the absence of a specific ligand. This basal KOP activation leads to the release of G_βγ_ subunits, which inhibits Ca^2+^ channels. EtOH disrupts the plasma membrane organization, causing deformation of the cholesterol-enriched membrane domains that harbor KOP, thereby promoting the dissociation of KOP homodimers into monomers. This reduces the number of KOPs that can be activated by thermal fluctuations, thereby diminishing KOP-mediated Ca^2+^ channel inhibition. Consequently, Ca^2+^ influx is enhanced, as observed with lower concentrations of EtOH (1~80 mM). In contrast, higher EtOH concentrations (>100 mM) cause significant distortion of the plasma membrane organization, leading to inhibition of Ca^2+^ channels.

Under treatment with NTX, larger nanoscale KOP-harboring clusters are being formed and the population of KOP homodimers increases. NTX also exerts its antagonistic effect through its action on KOP, which results in the enhancement of Ca^2+^ influx. These NTX effects are sustained even under combined treatment with EtOH. Under combined treatment, both NTX and EtOH affect the KOP-harboring-cholesterol-enriched membrane domains, as observed in our previous study [8]. Under combined treatment with EtOH, cholesterol-enriched membrane domains of intermediate size form, and the KOP homodimer population is still decisive, albeit lower than under treatment with NTX alone. As a result, the Ca^2+^ influx remains the same in the whole EtOH concentration range. In other words, the EtOH-induced enhancement and distortion of Ca^2+^ influx are suppressed by NTX. Considering the lipid dynamics/membrane fluidity and KOP clustering, NTX shows a protective effect against EtOH, possibly through the retention of membrane-anchored actin fibers and membrane domains.

Comparative studies were performed using the known KOP antagonist LY2444296 that is a homolog of JNJ-67953964 (a.k.a. CERC-501 and LY2456302) and a NTX-related agent, and nalfurafine, a KOP agonist recently introduced for the treatment of itch (as developed in patients receiving opiates chronically) [66]. We confirm that nalfurafine, like the natural ligand DynA, induces KOP internalization (Fig. S7). But, unlike DynA (Fig. S2) nalfurafine leads to the formation of large cytoplasmic vesicles with a clear KOP-eGFP harboring rim (Fig. S7), that are significantly larger (can be readily distinguished by confocal microscopy) than the typically observed KOP-eGFP trafficking vesicles the size of which is below/at the resolution limit defined by the diffraction of light. The observed activity of the LY2444296 confirmed KOP antagonism and as is shown here, NTX and LY2444296 share binding sites (Fig. S8).

It can be noted that KOP has been identified as one of the strongest genetic linkages in major depressive disorder along with dopamine receptor 2 (D2R) [67]. It is noticeable that there is a striatonigral dynorphin pathway reciprocal to the classic nigrostriatal dopamine pathway [68, 69]. The close connection between two potentially relevant neurotransmitter systems may be an indication of a functional relationship. A recent “fast-fail” study of JNJ-67953964 in major depressive disorder showed activity in anhedonia [70]. It is now in Phase III trial as Aticaprant. Another chemically distinct KOP antagonist BTRX-335140, Navacaprant is in Phase II clinical trial in depression [71]. To our knowledge, there are so far no clinical studies of KOP antagonists in AUD. Our research using an alcohol-dependent rat model demonstrated that LY2444296 significantly reduced alcohol self-administration in both male and female rats. These findings suggest that KOP-selective antagonists could be promising candidates for developing medications to treat AUD [72].

## Concluding remarks

NTX, an analog of naloxone–a well-known opiate antidote in emergencies, was developed as a long-acting MOP antagonist to protect against further intoxication. Its activity in AUD, initially thought to be limited to its antagonistic action on MOP, is multifaceted, involving its potent antagonistic action on KOP, also shown here. Our data suggest that in addition to these systemic effects, alcohol also exerts its effects on KOP function at the cellular level. By changing KOP distribution between the lipid bilayer and nanoscale clusters within the plasma membrane, alcohol changes cellular KOP-mediated signaling. While NTX is blocking both constitutive and alcohol-induced KOP-mediated activity, it is important to note that it is much less active at KOP when alcohol is abound. This is an obvious caveat for NTX use in binge drinking (to intoxication), which is of medical concern and is very different from social alcohol use [73].

The interactions of alcohol, NTX and the opioid receptors, particularly MOP, have been studied with a variety of technologies [6, 74]. The current data illustrate that the KOP receptor and KOP-lipid interactions are also a relevant target.

## Supplementary information

Supplementary information

## Data Availability

Raw data can be provided for investigations from SO.
