# Supplementary material for: Naltrexone blocks alcohol-induced effects on kappa-opioid receptors in the plasma membrane
Source: Transl Psychiatry. 2024 Nov 24;14:477. doi: 10.1038/s41398-024-03172-8 (PMC11586411; doi:10.1038/s41398-024-03172-8)
Supplement: Supplementary file 1 — Supplementary information [file 41398_2024_3172_MOESM1_ESM.pdf]

Supplementary Information:
Naltrexone blocks alcohol-induced effects on kappa-opioid receptors in the plasma membrane

Sho Oasa, Erdinc Sezgin, Yuelong Ma, David A. Horne, Mihajlo D. Radmilović, Tijana Jovanović-Talisman, Rémi Martin-Fardon, Vladana Vukojević* and Lars Terenius*

*Corresponding authors

Fluorescence lifetime imaging microscopy (FLIM)

We used our in-house scanning-free instrument based on massively parallel Fluorescence Correlation Spectroscopy (mpFCS) and Fluorescence Lifetime Imaging Microscopy (FLIM) [1]. FLIM measurements were performed for 10 min to obtain less noisy FLIM curves. By fitting a one-component exponential decay curve to the FLIM curves recorded in the plasma membrane using our dedicated software, eGFP fluorescence lifetime (FL) was measured. The fitting equation is:

$$I(t) = I_{\mathrm{off}} + Aexp\left(-\frac{(t-t_0)}{\tau_{\mathrm{f}}}\right) \quad -(1),$$

where $I(t)$ denotes photon counts at time t, I_{off} is the offset due to background photon counts, A is the amplitude of the FLIM curve, and τ_{f} is the eGFP FL. In each cell, the average eGFP FL was calculated from 6~8 independent positions in the plasma membrane.

To construct the dose-response curves based on eGFP FL measurements, we acquired for each treatment condition the average eGFP FL from measurements on ≥ 9 cells. Dose-response data were fitted using the Origin Data Analysis and Graphing Software (OriginLab Corporation, USA) and the following fitting equation:

$$FL = FL_{\mathrm{min}} + \frac{(FL_{\mathrm{max}} - FL_{\mathrm{min}})}{(1 + 10^{p\times(EC_{50}-x)})} \quad -(2),$$

where FL_{max} and FL_{min} denote the largest and the smallest FL value, respectively. p is an allosteric factor, and the EC_{50} value is the half-maximal effective concentration, *i.e.*, the concentration of the compound required to change by 50 % the eGFP FL.

Confocal Laser Scanning Microscopy (CLSM) imaging and Fluorescence Correlation Spectroscopy (FCS)

Confocal Laser scanning microscopy (CLSM) imaging and FCS measurements were performed using the LSM880 (Carl Zeiss) microscope system equipped with a 405 nm laser, 488 nm Ar-ion laser, 633 nm He-Ne laser, and a water immersion objective lens (C-Apochromat, 40×, 1.2 N.A., Corr, Carl Zeiss), a gallium arsenide phosphide (GaAsP) detector and photomultiplier tube (PMT) detectors. eGFP and Abberior Star Red/Alexa Fluor 633 were excited using the 488 nm laser and 633 nm laser, respectively. The pinhole size was adjusted to 1 AU (40 μm). Fluorescence of eGFP and Abberior Star Red or Alexa Fluor 633 was simultaneously acquired. For this purpose, the fluorescence signal was split using a diffraction grating, and directed to the GaAsP detector (eGFP signal) and the PMT detector (Abberior Star Red or Alexa Fluor 633 signal). The detection wavelength was 500-570 nm for eGFP and 650-750 nm for Abberior Star Red or Alexa Fluor 633). Time-resolved fluctuations in fluorescence intensity were recorded in the plasma membrane in 10 consecutive series, each lasting 20 s.

For Ca^{2+} imaging, Ca^{2+}-unbound Fura Red and eGFP were excited using the 488 nm laser. The Ca^{2+}-bound Fura Red was excited using the 405 nm laser. To minimize crosstalk, multi-track mode was used. The optical setting was as follows: for Ca^{2+}-unbound Fura Red, the excitation wavelength was 488 nm, 600 μm pinhole and 650-740 nm detection; for Ca^{2+}-bound Fura Red the excitation wavelength was 405 nm, 90 μm pinhole and 650-740 nm detection; for KOP-eGFP, the excitation wavelength was 488 nm, 90 μm pinhole, 493-580 nm detection.

Time-lapse CLSM imaging was used for the K^+ stimulation series. Each time series was recorded for 10 min after K^+ stimulation.

For Fluorescence Recovery After Photobleaching (FRAP) analysis of the immobile KOP-eGFP fraction in the plasma membrane, fluorescence signals were continuously recorded with 280 ms frame time at 64×256 pixels of region of interests. Photobleaching was performed using at full power the 488 nm laser. FRAP was monitored for 40 s thereafter.

Membrane fluidity *via* General Polarization (GP) analysis

Untransfected wild type PC12 cells were used in general polarization (GP) analysis, because eGFP produces an inseparable cross-talk signal with NR12S. Cells were stained using 1 μg/mL NR12S

in PBS for 5 min before fluorescence imaging [2]. Free NR12S was washed out twice with phenol red-free medium.

Spectral imaging was performed using the LSM780 confocal microscope (Carl Zeiss) equipped with a 32-channel GaAsP array detector. NR12S was excited with the 488 nm laser, and the fluorescence signal was collected at 494 and 691 nm.

The images were analyzed with the freely available Fiji/ImageJ plugin (NIH), as described in Sezgin et. al. [3]. GP values were calculated as follows:

$$GP = \frac{I_{570} - I_{650}}{I_{570} + I_{650}} - (3),$$

where I_{570} and I_{650} are fluorescence intensities at 570 nm and 650 nm, respectively.

Calcium imaging

To analyze the functional activity recorded after the treatment with antagonist and EtOH at 37 °C, time-lapse fluorescence imaging was performed using the LSM880 microscope system as described in the microscopy section above. Altogether 55 fluorescence images were collected in 10 min. The first 5 images were collected before the K^+ stimulation, and then 50 images were collected after addition of 40 µL stimulation buffer to 200 µL of solution in each chamber of the 8-well chambered coverglass with PC12/KOP-eGFP cells. The stimulation buffer was composed of 2.2 mM $CaCl_2$ and 100 mM KCl in Dulbecco's phosphate-buffered saline without calcium and magnesium (Thermo Fisher Scientific).

To account for photobleaching of Fura Red during time-lapse fluorescence imaging, we calculated the Fura Red ratio (FR):

$$FR(t) = \frac{I(t)_{\text{Ca-bound}}}{I(t)_{\text{Ca-unbound}}} - (4),$$

where $I(t)$ is the fluorescence intensity of Ca^{2+}-bound and Ca^{2+}-unbound Fura Red. To quantify the fold change after K^+ stimulation, the Fura Red ratio was normalized by the average Fura Red ratio before the stimulation.

FCS data analysis

Data acquired by FCS was analyzed using the ZEN software (Carl Zeiss). Autocorrelation curves (ACCs) were generated by plotting as a function of lag time (τ) the autocorrelation function the green, $G_G(\tau)$, or the red, $G_R(\tau)$, channel that were calculated as follows:

$$G_i(\tau) = \frac{\langle I_i(t) \cdot I_i(t+\tau)\rangle}{\langle I_i(t)\rangle^2} \quad - (5),$$

where i indicates the green (G) or the red channel (R), τ denotes the lag time, and I_i is the fluorescence intensity in the green or the red channels.

The autocorrelation curves were analyzed by fitting, using a two-component model for 2D diffusion for KOP-eGFP and 3D diffusion for ASR-DOPE or fNTX, as follows:

$$G_{\mathrm{KOP}}(\tau) = 1 + \left(1 + \frac{F_{\mathrm{t,KOP}} \cdot e^{\frac{\tau}{\tau_{\mathrm{t,KOP}}}}}{1 - F_{\mathrm{t,KOP}}}\right) \cdot \frac{1}{N_{\mathrm{KOP}}} \cdot \left[F_{1,\mathrm{KOP}} \cdot \left(\frac{1}{1 + \frac{\tau}{\tau_{\mathrm{D1,KOP}}}}\right) + (1 - F_{1,\mathrm{KOP}}) \cdot \left(\frac{1}{1 + \frac{\tau}{\tau_{\mathrm{D2,KOP}}}}\right)\right] - (6)$$

$$G_R(\tau) = 1 + \left(1 + \frac{F_{\mathrm{t},R} \cdot e^{\frac{\tau}{\tau_{\mathrm{t},R}}}}{1 - F_{\mathrm{t},R}}\right) \cdot \frac{1}{N_R} \cdot \left[F_{1,R} \cdot \left(\frac{1}{1 + \frac{\tau}{\tau_{\mathrm{D1},R}}}\right) \cdot \left(\frac{1}{1 + \frac{\tau}{S_R^2 \cdot \tau_{\mathrm{D1},R}}}\right)^{\frac{1}{2}} + (1 - F_{1,R}) \cdot \left(\frac{1}{1 + \frac{\tau}{\tau_{\mathrm{D2},R}}}\right)\right] - (7),$$

where: F_t is the average fraction of fluorescent molecules in the triplet state; τ_t is the average relaxation time of the triplet state; N is the average number of fluorescent molecules in the effective volume elements; F_1 and F_2 are the relative molar fractions of the first and the second component, respectively (F_1+F_2 = 1); τ_{D1} and τ_{D2} are the average diffusion times of the first and the second component, respectively; S is the structure parameter ($S_i = z_i/w_i$).

To quantify the membrane-related diffusion time, diffusion time of the first component of Abberior Star Red-DOPE was fixed to the value measured in solution, because this component originates from the free-diffusion of the Abberior Star Red-DOPE in the cell culture medium.

Values of the lateral radius (w_i) and axial radius (z_i) were determined in calibration measurements using ATTO488 ($D_{ATTO488}$; 400 $\mu m^2 s^{-1}$) for measurements in the green channel and Cy5 (D_{Cy5}; 360 $\mu m^2 s^{-1}$) for measurements in the red channel [4]:

$$w_i = \sqrt{4 \cdot D_i \cdot \tau_{D,i}} - (8)$$

$$z_i = w_i \cdot S_i - (9),$$

where i indicates the green (G) or red (R) channel; D_G and D_R are diffusion coefficients of ATTO488 and Cy5, respectively; $\tau_{D,G}$ and $\tau_{D,R}$ are the diffusion times of ATTO488 and Cy5, respectively. The diffusion coefficients of KOP-eGFP and Abberior Star Red-DOPE were calculated from the diffusion time of each molecule and the lateral radius determined as described above.

The average numbers of KOP-eGFP (N_{KOP}), ASR-DOPE (N_{DOPE}) is obtained from the fitting analysis above. To quantitatively characterize homodimerization of KOP-eGFP and lipid organization, counts per particle (CPP) were calculated as:

$$CPP_i = \frac{I_i}{N_i} - (10),$$

where i denotes KOP-eGFP or ASR-DOPE, I is the average fluorescence intensity, and N is the number of molecules from fitting analysis.

Total Internal Reflection Fluorescence Microscopy-integrated Fluorescence Correlation Spectroscopy (TIR-FCS)

TIR-FCS measurements [5-7] were performed using the Nikon Eclipse TE2000-E equipped with 488 nm laser, Dichroic mirrors (480/±10 nm), an oil immersion objective lens (Apo TIRF 100× N.A. 1.49, Nikon), emission filter (510-560 nm) and an Electron Multiplying Charge Coupled Device (EMCCD) camera, iXon Ultra 897. KOP-eGFP in the plasma membrane of U2OS cells was selectively excited using TIRF illumination of the 488 nm laser. Fluorescence signal was recorded by EMCCD camera with 1 ms frame time at 20×20 pixels of region of interests in the plasma membrane. Measurements last 50 s. Time series of fluorescence images were saved as TIF format.

All data were analyzed by free Image J plugin, imaging FCS 1 52 provided by Dr. Thorsten Wohland group (https://www.dbs.nus.edu.sg/lab/BFL/imfcs_image_j_plugin.html). Briefly, fluorescence intensity fluctuations were corrected by 4th polynomial equation to account for photobleaching. To obtain better autocorrelation curves for fitting analysis, 2×2 pixels were binned. To determine average diffusion coefficients in each cell, autocorrelation curves were fitted with one-component diffusion model given that the first component is shorter than 1 ms and cannot be seen here due to lower temporal resolution as compared to conventional FCS. Same Image J plugin was also used for diffusion law analysis [8].

FRAP data analysis

Time series of fluorescence intensity of KOP-eGFP were measured in the photobleaching region and in a reference region of interest, where deliberate photobleaching of the signal was not performed. Relative fluorescence intensity (RFI) was calculated as follows:

$$RFI(t)_{\text{KOP}} = \frac{FI(t)_{\text{bleach}}}{FI(t)_{\text{ref}}}, -(11)$$

where FI_{bleach} and FI_{ref} are fluorescence intensities in the photobleached and the reference regions, respectively. RFI was further re-scaled using RFI before photobleaching and the minimum value after photobleaching:

$$RFI(t)_{\text{re-scale, KOP}} = \frac{RFI(t)_{\text{KOP}} - RFI_{\text{min,KOP}}}{RFI_{\text{before,KOP}} - RFI_{\text{min,KOP}}}, -(12)$$

where $RFI_{min,KOP}$ and $RFI_{before,KOP}$ are relative fluorescence intensities before photobleaching and at the minimum after the photobleaching.

To determine the maximum recovery of the re-scaled RFI, which is corresponding to the mobile fraction of KOP-eGFP in the plasma membrane, the time series of the re-scaled RFI data was fitted using the following two component model:

$$RFI(t)_{\text{re-scale,KOP}} = BG + F_m \cdot \left\{F_{\text{m1}}\left[1 - e^{-\frac{t}{t1}}\right] + (1 - F_{\text{m1}}) \cdot \left[1 - e^{-\frac{t}{t2}}\right]\right\} - (13)$$

Here, BG is the background, F_m is the mobile fraction of KOP-eGFP in the plasma membrane, t_1 and t_2 are recovery times of each mobile component, and F_{m1} is the relative molar fraction, *i.e.* sub-fraction of the mobile component (F_{m1}+F_{m2} =1). Immobile fraction (F_{im}) was calculated as F_{im} = 1 - F_m.

Detailed procedure to synthesize naltrexone fluorescently labelled with Alexa Flour 633

A solution of naltrexone hydrochloride (Thermo Scientific) was prepared by dissolving 10 mg (0.026 mmol) of naltrexone hydrochloride in 1.8 mL ethanol-water solution (2:1, v:v). The solution was stirred, and under stirring 21.7 mg (0.106 mmol) of O-(6-aminohexyl)hydroxylamine dihydrochloride (ChemSpace) was added. The reaction between the components was allowed to proceed for 2 days at room temperature and under stirring [9]. After 2 days, the stirring was

stopped, the aqueous phase was collected and diluted with water for prep-RP-HPLC purification (Agilent 1200 prep system, Agilent 5 prep-C18 150 X 21.2 mm column, mobile phase A: 0.1% TFA in water, mobile phase B: 0.1% TFA in acetonitrile. Gradient 5% to 20% buffer B in 10 min, flow rate 20 mL/min). Eluent was combined and lyophilized to obtain naltrexone-C6-amine 16 mg as TFA salt and characterized by LC-mass (Agilent InfinityLAb LC/MSD system, Eclipse plus C18 column, 4.6 X 100 mm, 3.5 um, Gradient 5% to 35% buffer B in 20 min, flow rate 1 mL/min, retention time 9.8 min), purity 99%, expected mass [M+H]+ 456.3, observed [M+H]+ 456.3.

Naltrexone-C6-amine (1.3 mg) was dissolved in 1.5 mL acetonitrile-water (1:2, v:v) and 0.65 uL of diisopropylethanolamine was added to adjust the pH to pH 7-8. Subsequently, 1mg of Alexa Flour 633 carboxylic acid, succinimidyl was mixed at room temperature for 1 h, then purified by semi-prep RP-HPLC (Agilent 1200 prep system, Gemini-NX 5um C18, 250 X 10.0 mm column, mobile phase A: 0.1% TFA in water, mobile phase B: 0.1% TFA in acetonitrile. Gradient 25% to 55% buffer B in 20 min, flow rate 5 mL/min). Eluent was combined and lyophilized to obtain naltrexone-C6-Alexa Fluor 633. Product was characterized by LC-mass mass (Agilent InfinityLAb LC/MSD system, Eclipse plus C18 column, 4.6 X 100 mm, 3.5 um, Gradient 20% to 50% buffer B in 20 min for naltrexone-C6-Alexa Fluor 633, running speed 1 mL/min, retention time 10.2 min), purity 96.4%, observed mass [M+2H]2+ 681.7, the structure of Alexa flour 633 is not disclosed but the mass is within the expected range. Quantity of Alexa dye was determined by specific UV molar extinction coefficient (cm-1M-1) at 159,000 for Alexa flour 633.

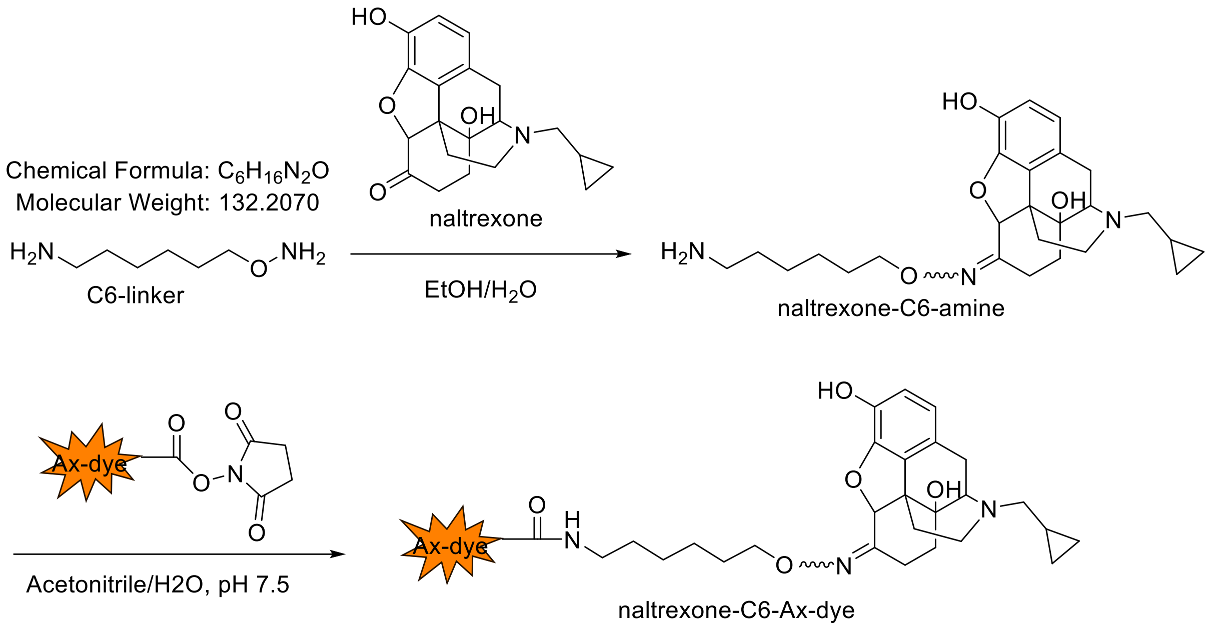

Figure S1. Schematic drawing of key steps in the synthesis of naltrexone fluorescently labelled with Alexa Fluor 633 (fNTX). To generate the fluorescent derivative of NTX that can bind to KOP, a long linker between KOP and the fluorescent moiety was chosen. This is based on the finding by X-ray crystallography using the KOP antagonist JDTic, which revealed that the KOP binding pocket is deeply inserted into the KOP molecule [Wu et al. Structure of the human κ-opioid receptor in complex with JDTic. Nature. 2012 485(7398):327-32]. Synthesis details are provided in the Supplementary Information text, above.

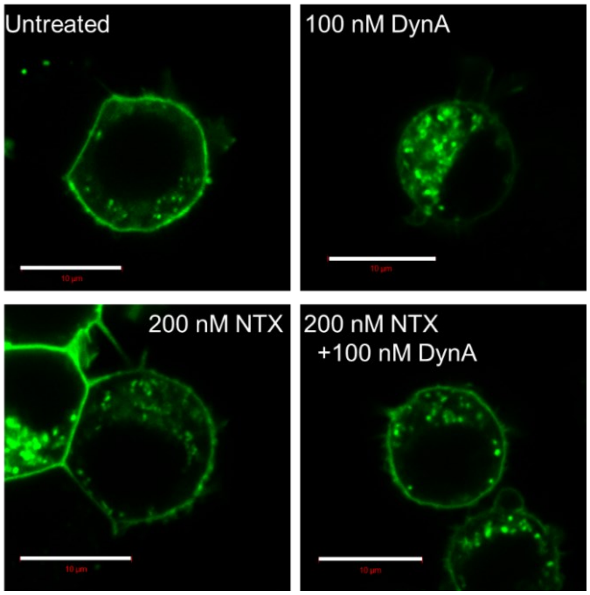

Figure S2. Dynorphin A (DynA) induced KOP-eGFP internalization and naltrexone (NTX) blocked DynA-induced internalization in PC12/ KOP-eGFP cells. Typical confocal fluorescence microscopy images of live PC12/KOP-eGFP cells show the subcellular localization of KOP-eGFP in untreated cells (upper, left); after 30 min treatment with 100 nM DynA (upper, right); after 30 min treatment with 200 nM NTX (lower, left); and after 30 min treatment with 200 nM NTX+100 nM DynA (lower, right). Scale bar: 10 µm.

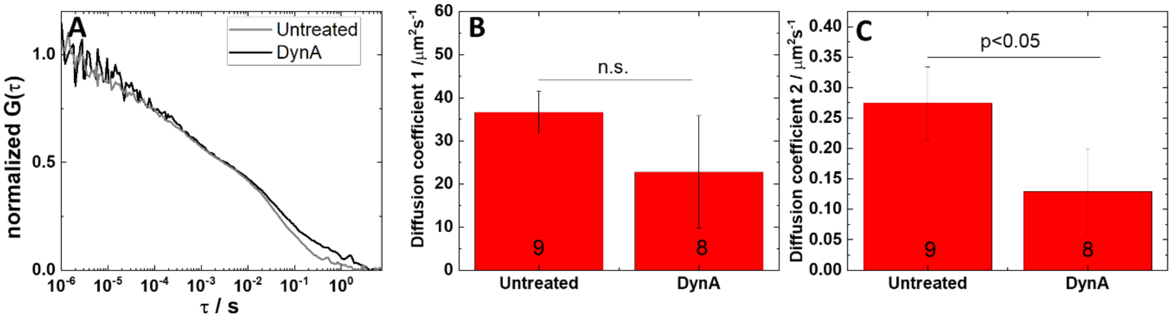

Figure S3. Dynorphin A (DynA) binding to KOP-eGFP slows down KOP-eGFP lateral diffusion in the plasma membrane of PC12/KOP-eGFP cells. (A) Autocorrelation curves normalized to the same amplitude, $G(\tau) = 1$ at $\tau = 10$ µs, recorded in untreated cells (grey) and cells treated with 100 nM DynA for 30 min (black). Slow diffusion component ($\tau_{D2} \approx 300$ ms) showed a statistically significant lowering of KOP-eGFP lateral diffusion following treatment with 100 nM DynA (C), whereas statistically significant difference in the fast diffusion component ($\tau_{D2} \approx 1$ ms) was not observed (B). **(B)** Diffusion coefficient of the fast-diffusion component. **(C)** Diffusion coefficient of slow-diffusion component. The number of analyzed cells is shown at the bottom of each bar. The two-tailed Student's t-test was used for statistical analysis.

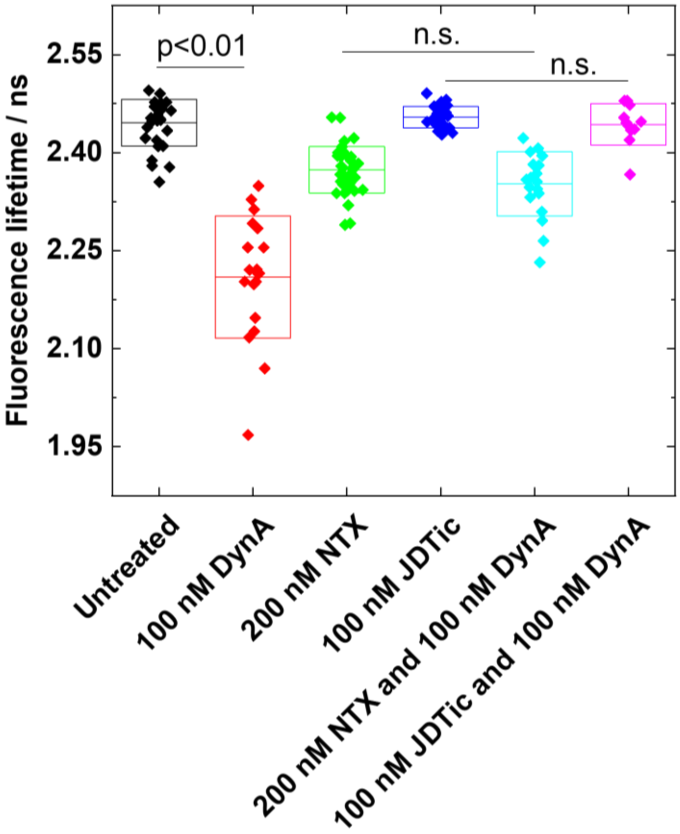

Figure S4. DynA-induced changes in eGFP fluorescence lifetime could be efficiently blocked by KOP antagonists NTX and JDTic. PC12/KOP-eGFP cells treatment with 100 nM DynA decreased eGFP fluorescence lifetime (FL) due to DynA binding to KOP-eGFP. This decrease in eGFP FL was efficiently blocked by naltrexone (NTX) and the KOP-selective antagonist JDTic. Black: untreated, Red: 100 nM DynA, Green; 200 nM NTX, Blue: 100 nM JDTic, Light blue: 200 nM NTX+100 nM DynA, Purple: 100 nM JDTic+100 nM DynA. Statistical analysis was performed using two-tailed Student's t-test. Lack of statistically significant difference (n.s.) indicates $p > 0.05$.

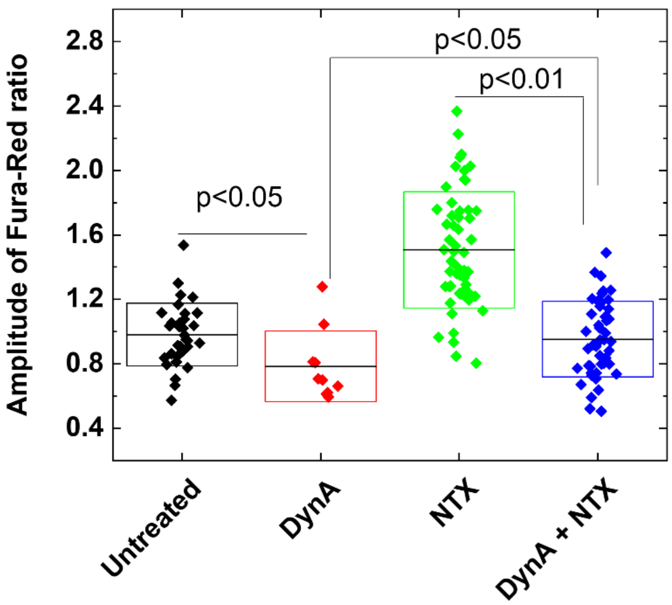

Figure S5. Naltrexone (NTX) blocks Dynorphin A (DynA)-induced effects on Ca^{2+} influx. Black: untreated, Red: 100 nM DynA, Green: 200 nM NTX, Blue: 200 nM NTX+100 nM DynA. Statistical analysis was performed using the two-tailed Student's t-test.

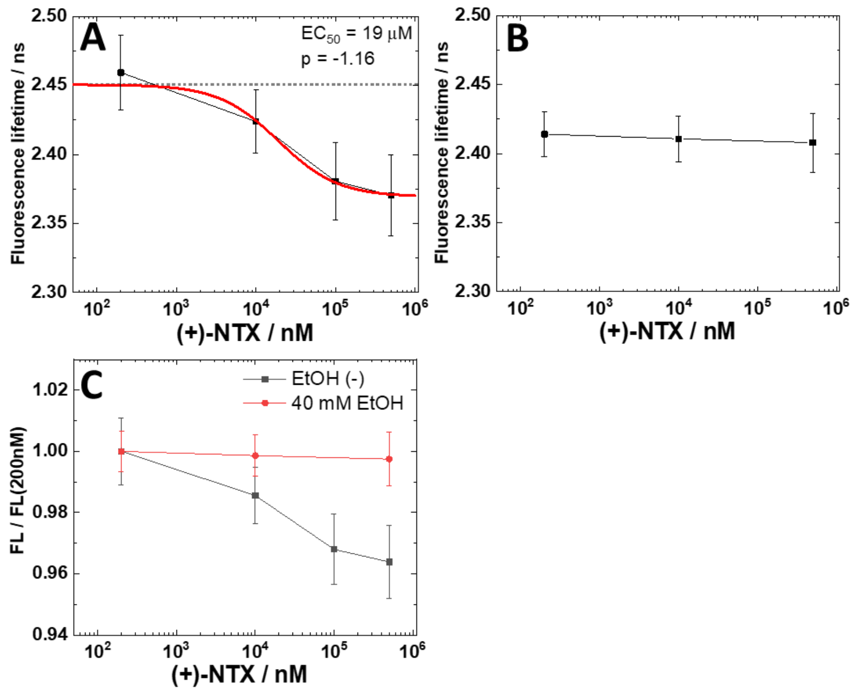

Figure S6. EtOH disrupted the NTX enantiomer (+)-NTX binding to KOP-eGFP in the plasma membrane. (A) (+)-NTX dose-response curve. Best fit of the dose-response curve (red) yielded 19 μM and -1.16 for the EC_{50} value and allosteric factor (p), respectively. **(B)** Dose-response curve for treatment with (+)-NTX + 40 mM EtOH. **(C)** eGFP FL normalized by eGFP FL measured for treatment with 200 nM (+)-NTX without or with 40 mM EtOH. Black: without EtOH, Red: with 40 mM EtOH.

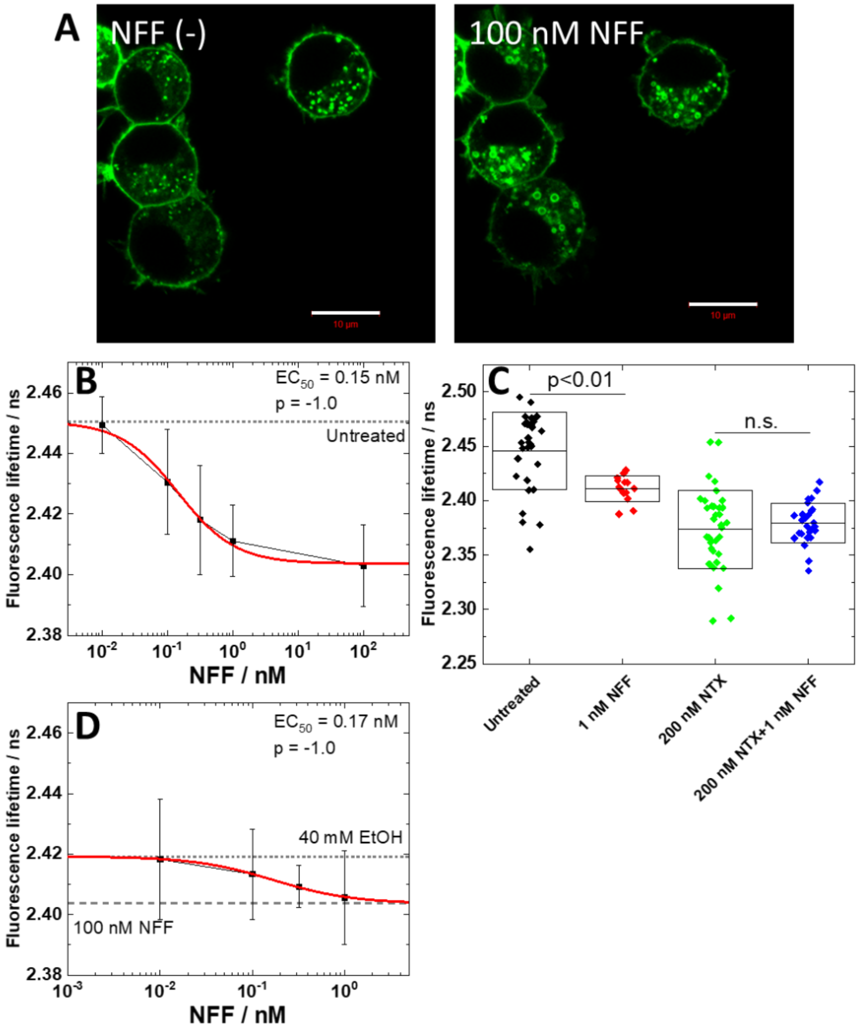

Figure S7. Nalfurafine (NFF) binding to KOP is not affected by EtOH. (A) Confocal fluorescence microscopy images of untreated PC12/KOP-eGFP cells (left, NFF(-)) and after 30 min treatment with 100 nM NFF (right). KOP-eGFP internalization is reflected by both, an increase in intracellular fluorescence and a decrease in plasma membrane-associated fluorescence. Scale bar: 10 μm. **(B)** Dose-response curve for NFF determined by FLIM. Best fit of the dose-response curve (red) yielded 0.15 nM and -1.0 as EC_{50} and allosteric factor (*p*), respectively. Grey dotted line: average eGFP FL in untreated cells. **(C)** Excessive amount of naltrexone (NTX) blocked NFF effect on eGFP FL. Black: untreated, Red: 1 nM NFF, Green: 200 nM NTX, Blue: 200 nM NTX+1 nM NFF. Statistical analysis was performed using the two-tailed Student's t-test. Lack of statistically significant difference (n.s.) indicates $p > 0.05$. **(D)** Dose-response curve for NFF + 40 mM EtOH. Best fit of dose-response curve yielded 0.17 nM and -1.0 as EC_{50} and allosteric factor (*p*), respectively. Grey dotted line: average FL in the presence of 40 mM EtOH, Grey dashed line: average FL under the 100 nM NFF.

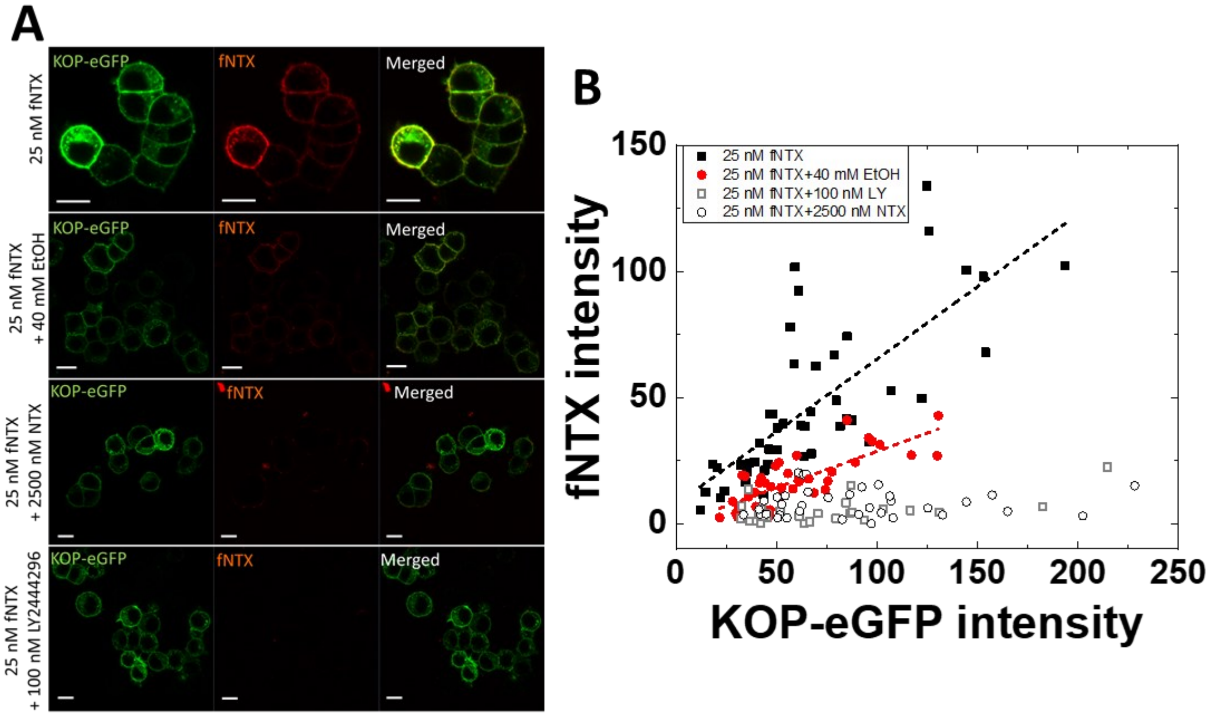

Figure S8. EtOH and KOP antagonists block fNTX binding to KOP in the plasma membrane. (A) Confocal fluorescence microscopy images of live PC12/KOP-eGFP cells (green) incubated under different conditions with Alexa633-labeled naltrexone (fNTX, red). *First row*: KOP-eGFP and fNTX localize in the plasma membrane in PC12/KOP-eGFP cells treated with 25 nM fNTX. *Second row:* fNTX binding to PC12/KOP-eGFP cells treated with 40 mM EtOH is significantly reduced. *Third row:* fNTX binding to PC12/KOP-eGFP cells is blocked by a large excess of non-labeled naltrexone (NTX). *Fourth row*: fNTX binding to PC12/KOP-eGFP treated with 100 nM KOP-selective antagonist LY2444296is efficiently blocked. Scale bar: 10 µm. **(B)** Scatter plot showing fluorescence intensity of fNTX in the plasma membrane of PC12/KOP-eGFP cells as a function of fluorescence intensity of KOP-eGFP. Black squares: treatment with 25 nM fNTX only. Red dots: treatment with 25 nM fNTX + 40 mM EtOH. Black rectangles: treatment with 25 nM fNTX + 100 nM LY2444296. Black circles: treatment with 25 nM fNTX + 2500 nM NTX. Best fit of linear regression (dashed lines) showed strong correlation between fNTX and KOP-eGFP fluorescence intensities with a slope of 0.75 for fNTX treatment (black dashed line), and 0.38 for 25 nM fNTX+40 mM EtOH (red dashed line). A close to zero slope was observed for treatments with 25 nM fNTX+2500 nM NTX (black circles) or 25 nM fNTX + 100 nM LY2444296 (black rectangles), showing that NTX and LY2444296 efficiently block fNTX binding to KOP-eGFP.

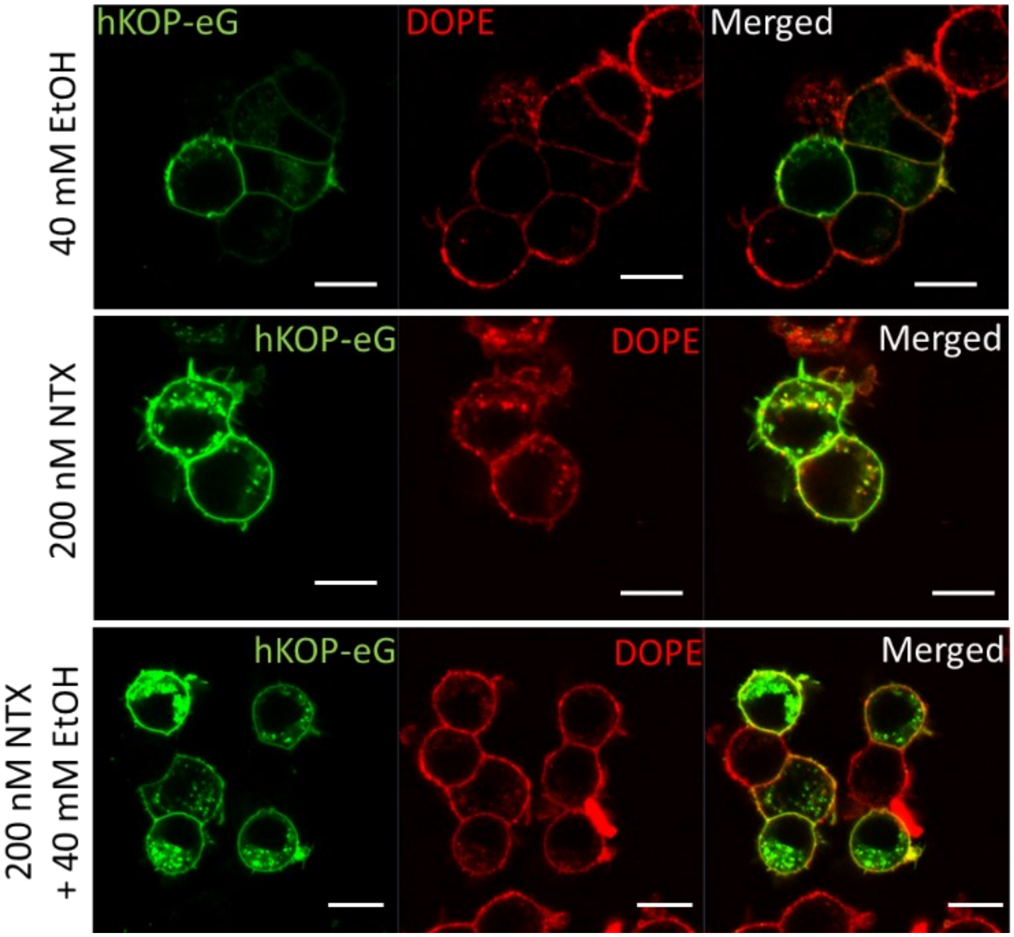

Figure S9. KOP-eGFP and Abberior Star Red-DOPE localization in untreated and treated PC12/KOP-eGFP cells. Confocal fluorescence microscopy images of PC12/KOP-eGFP cells (green) stained with Abberior Star Red-DOPE (red). *First row:* Cells treated with 40 mM EtOH. *Second row:* Cells treated with 200 nM NTX. *Third row:* Cells treated with 200 nM NTX+40 mM EtOH. Scale bar: 10 µm.

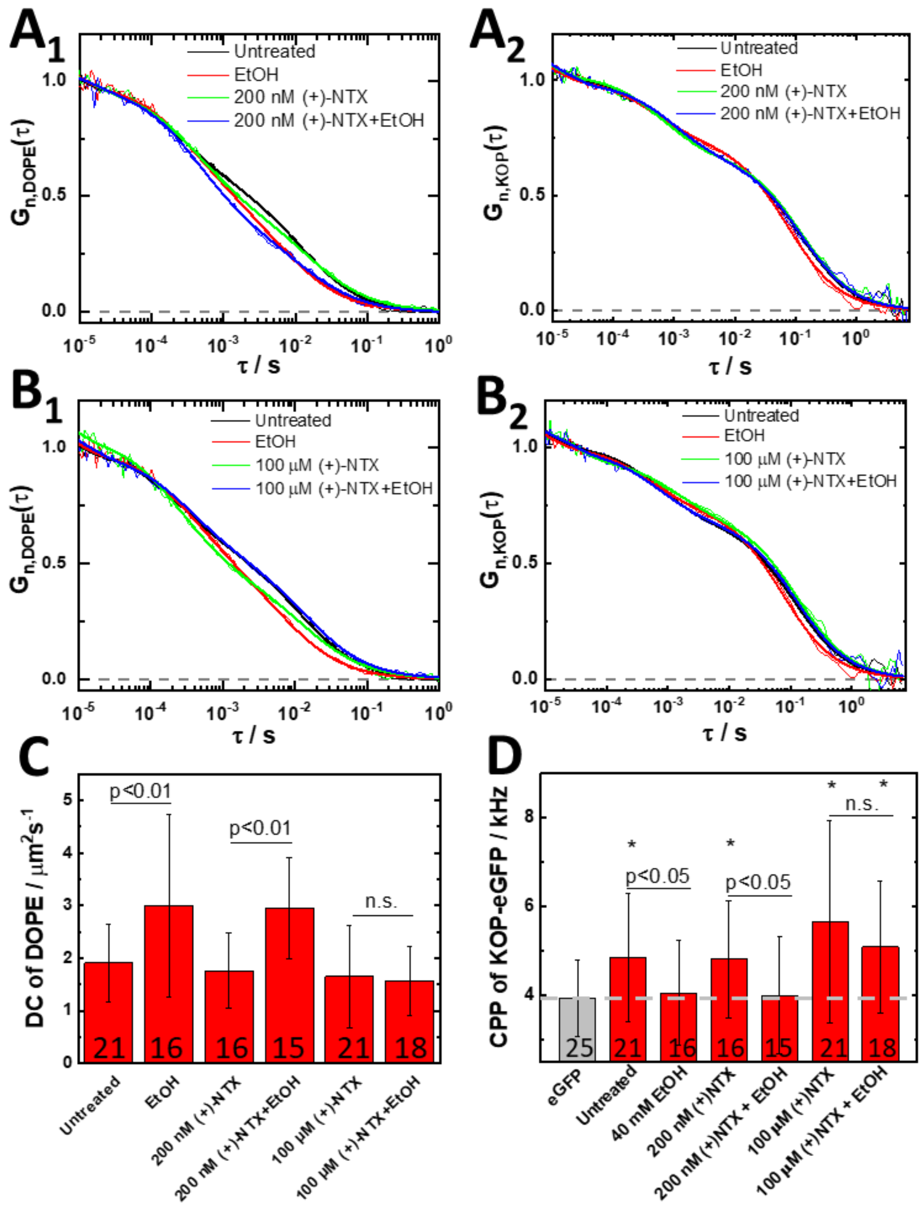

Figure. S10. The NTX enantiomer (+)-NTX cannot ward off EtOH-induced effects at same concentration as NTX. (A, B) Autocorrelation curves (ACCs) of Abberior Star Red-DOPE **(A_1, B_1)** and KOP-eGFP **(A_2, B_2)** under the treatment with 200 nM (A) and 100 µM (B) of (+)-NTX. The ACCs are normalized to the same amplitude, $G_n(\tau) = 1$ at $\tau = 10$ µs. **(C)** Diffusion coefficient (DC) of ASR-DOPE. **(D)** Apparent KOP-eGFP brightness, as reflected by counts *per* particle (CPP). Data reported in C and D are acquired for treatments with 40 mM EtOH, 200 nM or 100 µM (+)-NTX, or 200 nM + 40 mM EtOH or 100 µM (+)-NTX + 40 mM EtOH. The number of cells analyzed is indicated at the bottom of the bar. Statistical analysis was performed using the two-tailed Student's t-test. Asterisk (*) indicates significance, $p<0.01$, for comparisons against eGFP (dashed grey line).

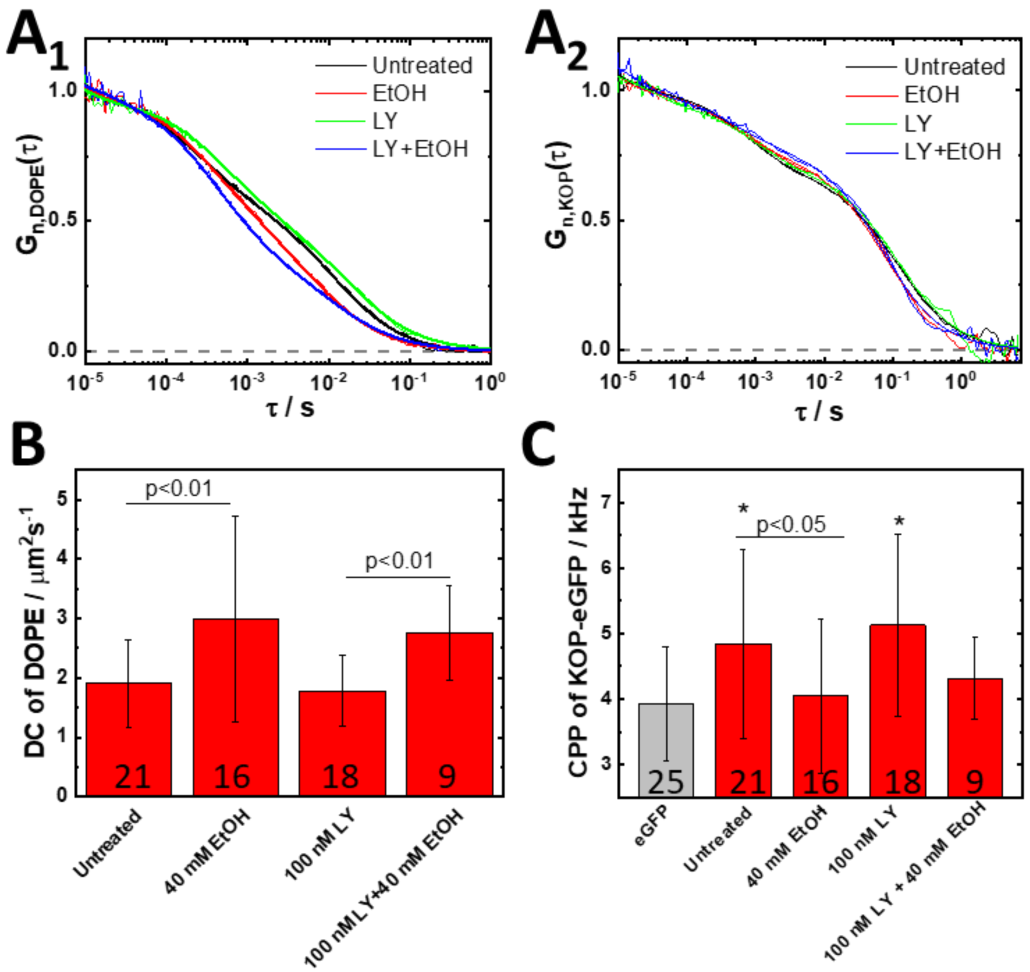

Figure S11. The KOP-specific antagonist LY2444296 does not alter the lipid dynamics and does not ward of EtOH-induced changes in the lipids' diffusion and KOP-eGFP lateral organization in the plasma membrane. (A) Autocorrelation curves (ACCs) of Abberior Star Red-DOPE **(A_1)** and KOP-eGFP **(A_2)** under the treatment with100 nM LY2444296 (LY) or 100 nM LY2444296 + 40 mM EtOH. ACCs are normalized to the same amplitude, $G_n(\tau) = 1$ at $\tau = 10$ µs. **(B)** Diffusion coefficient (DC) of ASR-DOPE. **(D)** Apparent KOP-eGFP brightness, as reflected by counts *per* particle (CPP). Data reported in C and D are acquired for treatments with 40 mM EtOH, 100 nM LY2444296 or 100 nM LY2444296 + 40 mM EtOH. The number of cells analyzed is indicated at the bottom of each bar. Statistical analysis was performed using the two-tailed Student's t-test. Asterisk (*) indicates significance, p<0.01, for comparisons against eGFP.

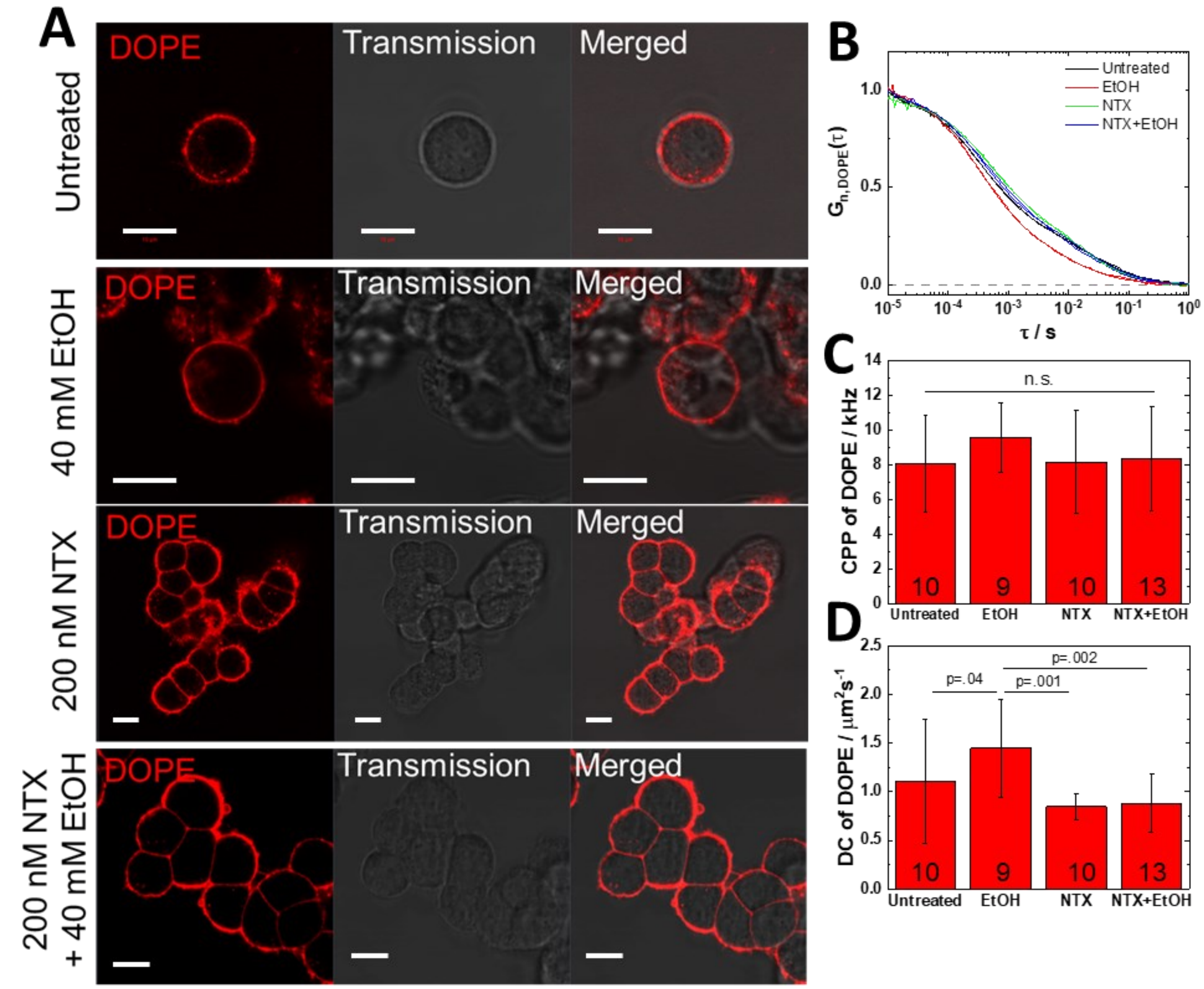

Figure S12. NTX wards off EtOH-induced effects on lipid dynamics in wild type PC12 cells. **(A)** Confocal fluorescence microscopy images of untransfected, wild type PC12 cells stained with ASR-DOPE (red). *First row:* Untreated cells. *Second row:* Cells treated with 40 mM EtOH. *Third row:* Cells treated with 200 nM NTX. *Fourth row:* Cells treated with 200 nM NTX+40 mM EtOH. Scale bar: 10 µm. **(B)** Autocorrelation curves (ACCs) of ASR-DOPE in the plasma membrane of live PC12 cells without and under the treatments tested. The ACCs are normalized to the same amplitude, $G_n(\tau) = 1$ at $\tau = 10$ µs. Black: Untreated. Red: 40 mM EtOH. Green: 200 nM NTX. Blue: 200 nM NTX + 40 mM EtOH. **(C)** ASR-DOPE brightness, as reflected by counts *per* particle (CPP) under each treatment. **(D)** Diffusion coefficient (DC) of ASR-DOPE in the plasma membrane (calculated from the longer characteristic decay time of the ACC) under each condition. The number of cells analyzed is indicated at the bottom of the bars.

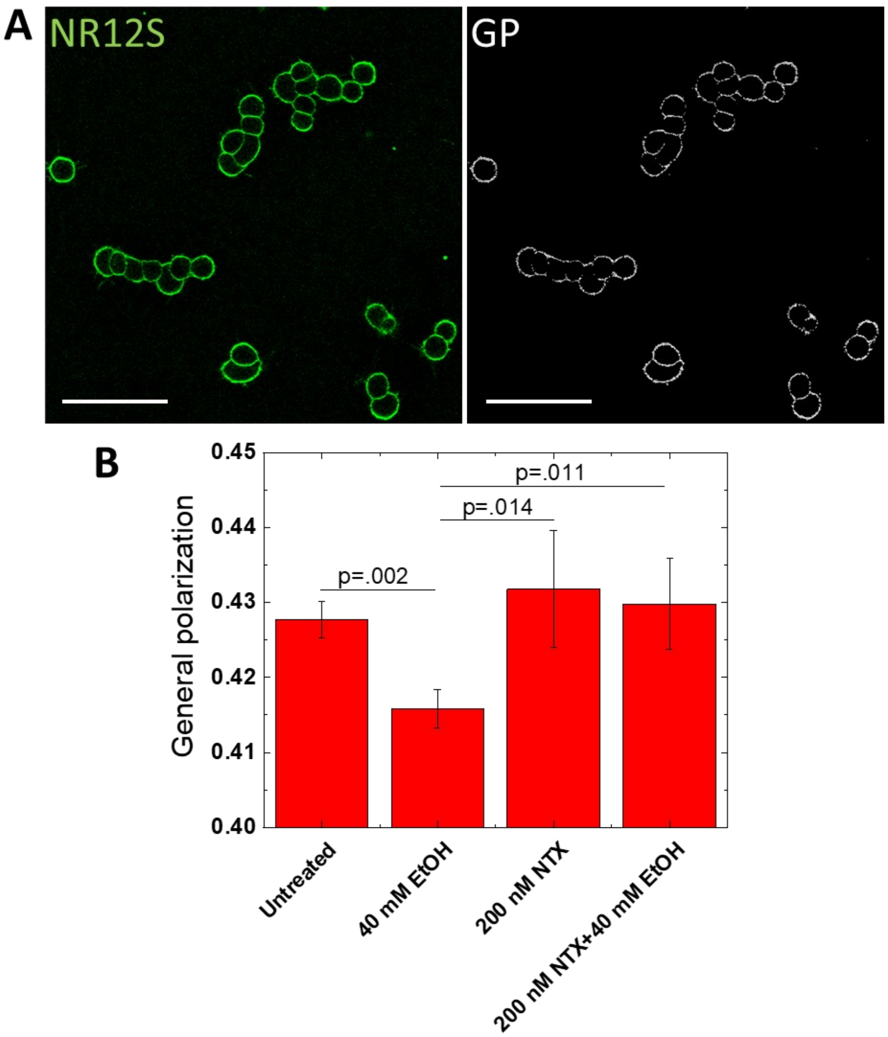

Figure S13. NTX wards off EtOH-induced effects on plasma membrane general polarization. (A) *Left:* Confocal fluorescence microscopy image of untransfected, wild type PC12 cells stained with NR12S. *Right:* General Polarization (GP) image computed from the spectral imaging data. Green: Total fluorescence intensity of NR12S observed in spectral imaging, Grey: GP image. Scale bar: 50 µm. **(B)** GP values in untreated cells and cells treated with 40 mM EtOH, 200 nM NTX or 200 nM NTX + 40 mM EtOH. Three independent experiments were performed, and the data are given as average ± standard deviation. Statistical analysis was performed using the two tailed Student's t-test.

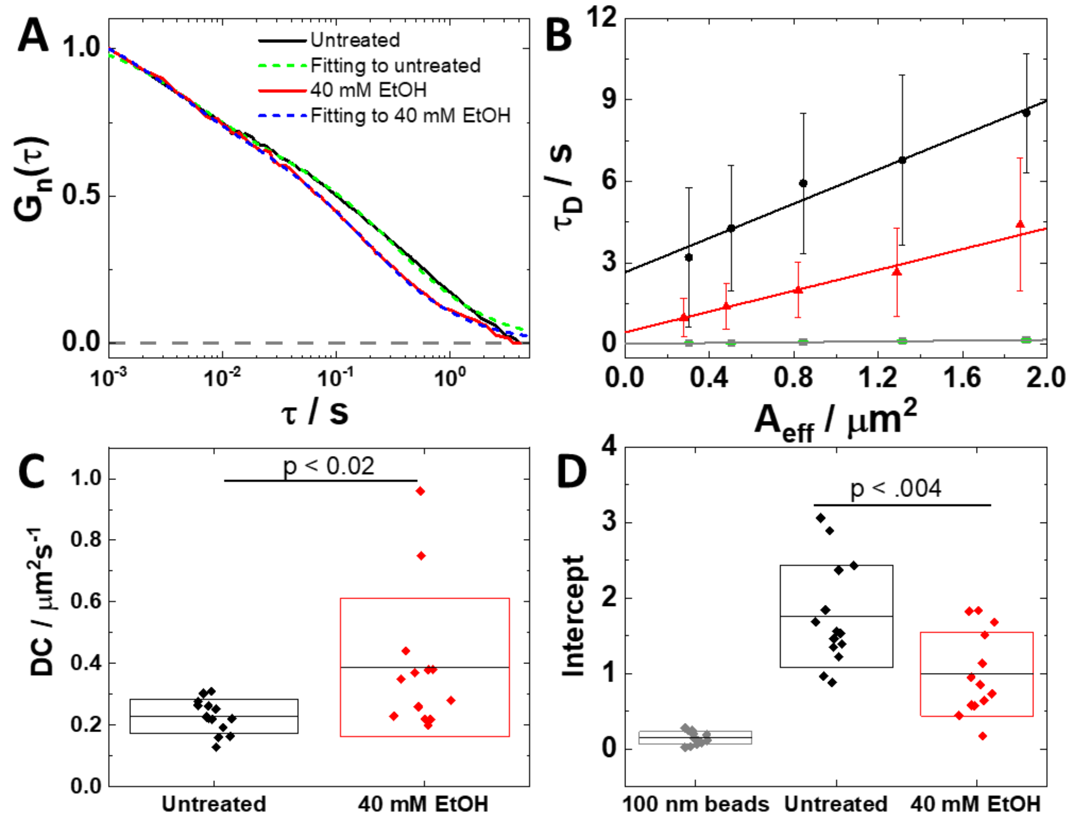

Figure S14. EtOH reduces KOP-eGFP confinement in membrane domains. (A) Autocorrelation curves (ACCs) of KOP-eGFP in untreated (black and fitted green dashed curve) and 40 mM EtOH-treated cells (red and fitted blue dashed curve). The ACCs are normalized to the same amplitude, $G_n(\tau) = 1$ at $\tau = 1$ ms. **(B)** Diffusion time (τ_D) as a function of the effective observation area (A_{eff}) for KOP-eGFP in untreated (black) and 40 mM EtOH treated cells (red). As a free diffusion control, the free diffusion of 100 nm fluorospheres in deionized water was observed (green). Positive intercept suggests that KOP-eGFP is confined in membrane domains. **(C)** Average diffusion coefficient (DC) of KOP-eGFP in untreated (black) and treated (red) cells. **(D)** The intercept from diffusion law analysis (D) for KOP-eGFP. Black: Untreated, Red: 40 mM EtOH, Grey: 100 nm fluorospheres in MQ water. Positive intercept was significantly reduced in the presence of 40 mM EtOH, suggesting that KOP-eGFP confined to membrane domains turns to free diffusion states via modulation of membrane environment by EtOH assessed by FLIM (Figure 1) and FCS with fluorescently labelled DOPE (Figure 3). To meet the requirement for TIR-FCS, which necessitates the use of adherent cells, diffusion law analysis was performed in U2OS cells transiently expressing KOP-eGFP.

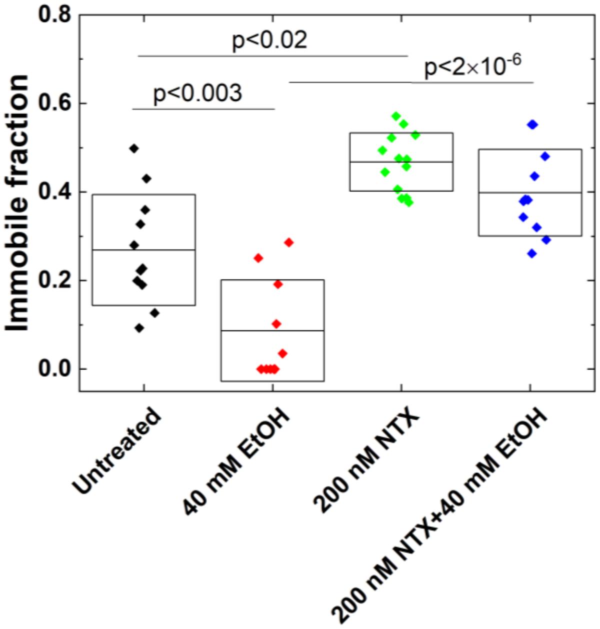

Figure S15. EtOH and NTX alter immobile fraction of KOP-eGFP. FCS quantitatively characterized only mobile component of KOP-eGFP, thus we also analyzed immobile fraction at the plasma membrane of PC12 cells by Fluorescence Recovery After Photobleaching (FRAP). Immobile fraction of KOP-eGFP was reduced in the presence of 40 mM EtOH (red), suggesting that a larger portion of KOP-eGFP molecules is in the more mobile pool. In contrast, NTX increased the fraction of KOP-eGFP molecules in the immobile pool (green) and blocked the effects of EtOH (blue). Statistical analysis was performed using the two-tailed Student's t-test.

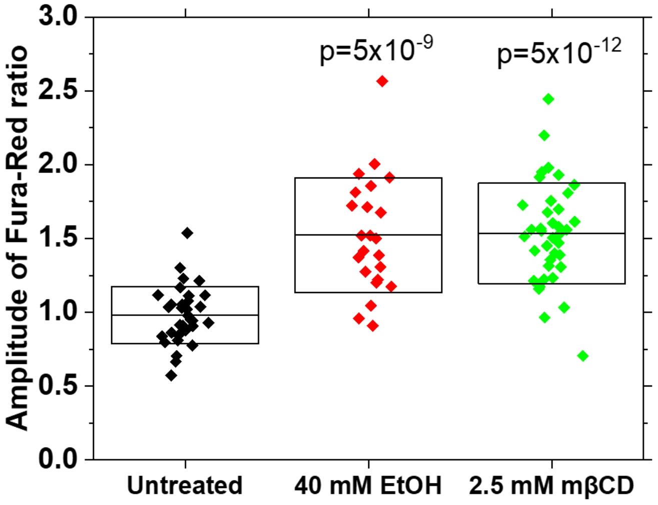

Figure S16. Ca^{2+} influx under cholesterol depletion. Average ± Standard deviation of amplitude of Fura Red ratio in PC12 cells expressing KOP-eGFP under the treatment with 40 mM EtOH and 2.5 mM mβCD. Black: Untreated, Red: 40 mM EtOH, Green: 2.5 mM mβCD. Statistical analysis was performed using the two-tailed Student's t-test. The *p* values given are against untreated cells.

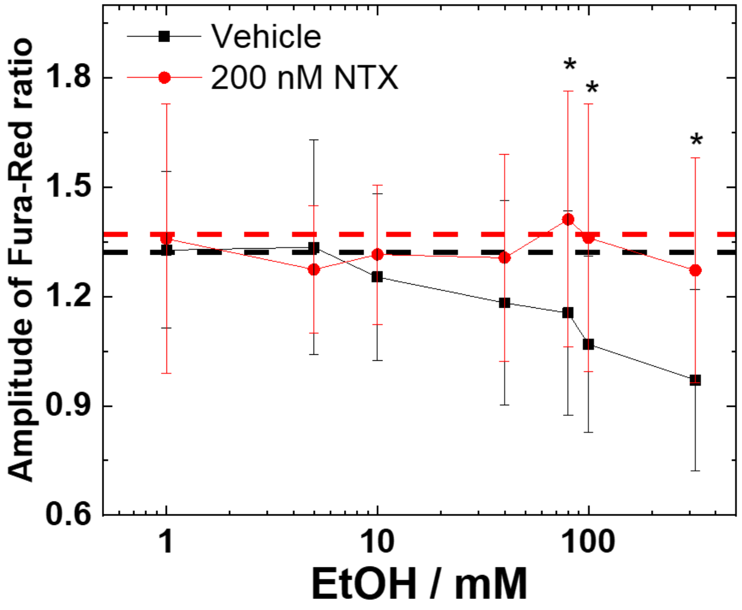

Figure S17. Ca^{2+} influx in untransfected wild type PC12 cells. Untransfected wild type PC12 cells were stained with Fura Red. Black: different dose of EtOH (1 mM to 320 mM), Red: 200 nM NTX + different dose of EtOH (1 mM to 320 mM). Black dashed line: average amplitude of Fura Red in Untreated cells. Red dashed line: average amplitude of Fura Red in cells treated with 200 nM NTX. Statistical analysis was performed using the two-tailed Student's t-test. Asterisk (*) indicates significance, $p<0.0002$, for comparisons between vehicle against 200 nM NTX for the same EtOH concentration.

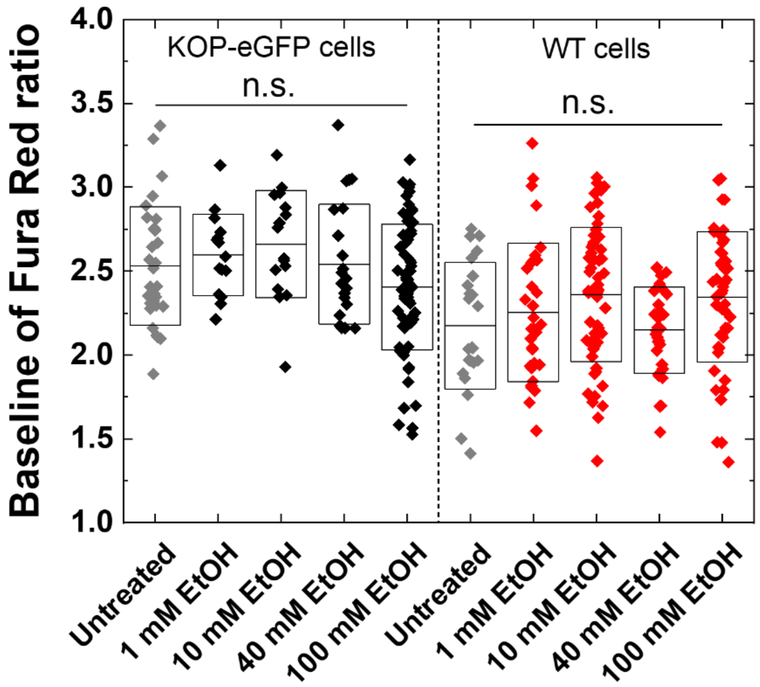

Figure S18. EtOH does not affect the baseline of the Fura Red ratio before K^+ depolarization. Acute EtOH treatment did not disrupt the intercellular Ca^{2+} ion homeostasis and did not induce endoplasmic reticulum (ER) stress and Ca^{2+} release from the ER. Grey: Untreated PC12/KOP-eGFP cells (left) and wild type PC12 cells (right). Black: 1 mM, 10 mM, 40 mM or 100 mM EtOH-treated PC12/KOP-eGFP cells. Red: 1 mM, 10 mM, 40 mM or 100 mM EtOH-treated PC12 cells. Statistical analysis was performed using ANOVA test with post hoc Turkey test. The lack of statistical significance is designated, n.s., $p > 0.05$.

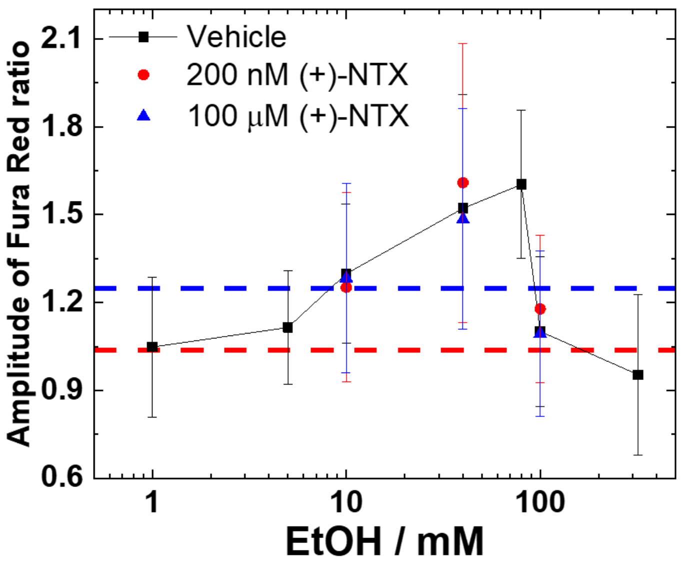

Figure S19. (+)-NTX has no effect on EtOH-modulated Ca^{2+} influx. PC12/KOP-eGFP treated with two different concentrations of (+)-NTX; 200 nM or 100 μM, which are lower and higher, respectively than the EC_{50} value measured by FLIM (19 μM). Black: different doses of EtOH alone. Red: 200 nM (+)-NTX with different doses of EtOH. Blue: 100 μM (+)-NTX with different doses of EtOH. Red dashed line: mean amplitude of the Fura Red ratio for treatment with 200 nM (+)-NTX. Blue dashed line: mean amplitude of Fura Red ratio for treatment with 100 μM (+)-NTX.

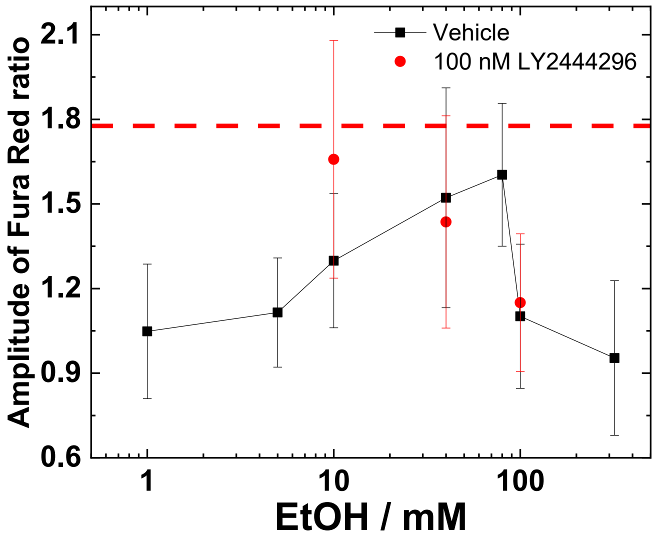

Figure S20. The KOP-specific antagonist LY2444296 highly affects Ca^{2+} influx at low EtOH concentrations (< 40 mM)**.** In PC12/KOP-eGFP cells treated with 100 nM LY2444296, a highly enhanced amplitude of Fura Red ratio is observed for lower (< 40 mM) EtOH concentrations, dropping down to the vehicle level for 100 mM EtOH. Black: different dose of EtOH alone. Red: 100 nM LY2444296 with different doses of EtOH. Red dashed line: mean amplitude of Fura Red ratio for treatment with 100 nM LY2444296 alone.

Supplementary References

1. Oasa S, Krmpot AJ, Nikolic SN, Clayton AHA, Tsigelny IF, Changeux JP et al. Dynamic Cellular Cartography: Mapping the Local Determinants of Oligodendrocyte Transcription Factor 2 (OLIG2) Function in Live Cells Using Massively Parallel Fluorescence Correlation Spectroscopy Integrated with Fluorescence Lifetime Imaging Microscopy (mpFCS/FLIM). *Anal Chem* 2021; 93: 12011-21.
2. Sezgin E, Schneider F, Zilles V, Urbancic I, Garcia E, Waithe D et al. Polarity-Sensitive Probes for Superresolution Stimulated Emission Depletion Microscopy. *Biophys J* 2017; 113: 1321-30.
3. Sezgin E, Waithe D, Bernardino de la Serna J and Eggeling C. Spectral imaging to measure heterogeneity in membrane lipid packing. *Chemphyschem* 2015; 16: 1387-94.
4. Kapusta P. Absolute Diffusion Coefficients: Compilation of Reference Data for FCS Calibration. *Application Note (PicoQuant) Available at https://www.picoquant.com/images/uploads/page/files/7353/appnote_diffusioncoefficients.pdf* 2010;
5. Macháň R and Wohland T. Recent applications of fluorescence correlation spectroscopy in live systems. *FEBS Letters* 2014; 588: 3571-84.
6. Sankaran J, Balasubramanian H, Tang WH, Ng XW, Röllin A and Wohland T. Simultaneous spatiotemporal super-resolution and multi-parametric fluorescence microscopy. *Nat commun* 2021; 12: 1748.
7. Bag N, Ng XW, Sankaran J and Wohland T. Spatiotemporal mapping of diffusion dynamics and organization in plasma membranes. *Methods Appl Fluoresc* 2016; 4: 034003.
8. Ng XW, Bag N and Wohland T. Characterization of Lipid and Cell Membrane Organization by the Fluorescence Correlation Spectroscopy Diffusion Law. *CHIMIA* 2015; 69: 112.
9. Cerri A, Almirante N, Barassi P, Benicchio A, Fedrizzi G, Ferrari P et al. 17β-O-Aminoalkyloximes of 5β-Androstane-3β,14β-diol with Digitalis-like Activity: Synthesis, Cardiotonic Activity, Structure−Activity Relationships, and Molecular Modeling of the Na+,K+-ATPase Receptor. *Journal of Medicinal Chemistry* 2000; 43: 2332-49.
